# Supplementary material for: Interplay of SLC33A1-dependent and -independent Golgi sialic acid O-acetylation in CASD1 catalysis
Source: Nat Commun. 2026 Apr 1;17:3156. doi: 10.1038/s41467-026-71333-y (PMC13043746; doi:10.1038/s41467-026-71333-y)
Supplement: Supplementary file 1 — Supplementary Information [file 41467_2026_71333_MOESM1_ESM.pdf]

Supplementary Figures

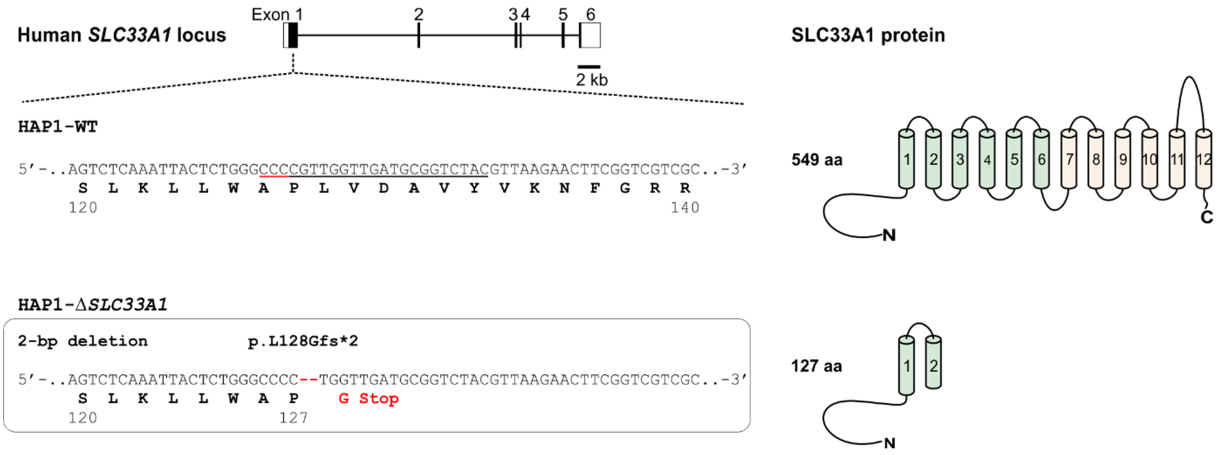

Supplementary Figure 1.

Genetic inactivation of *SLC33A1* in the human haploid cell line HAP1. Schematic representation of the human *SLC33A1* locus showing the target site in exon 1 used for CRISPR/Cas9-mediated inactivation of *SLC33A1* in HAP1 cells. Target sequence and protospacer adjacent motif (PAM) are underlined (black and red line, respectively). A 2-bp microdeletion found in the HAP1-Δ*SLC33A1* clone used in this study is depicted in red together with the resulting changes in the translation product. The frameshift mutation leads to a premature stop and the corresponding translation product, L128Gfs*2, encompasses only 127 of the original 549 amino acids (aa) spanning wild-type sequence. Inspection of the cryo-EM structure of SLC33A1 (PDB ID 9M0S)[1] revealed that the truncated translation product lacks TMH3 to TMH12 as shown schematically in comparison to the parental protein (right panel). Genetic alterations have been validated on genomic and transcript level by (RT-)PCR followed by Sanger sequencing of the obtained PCR product.

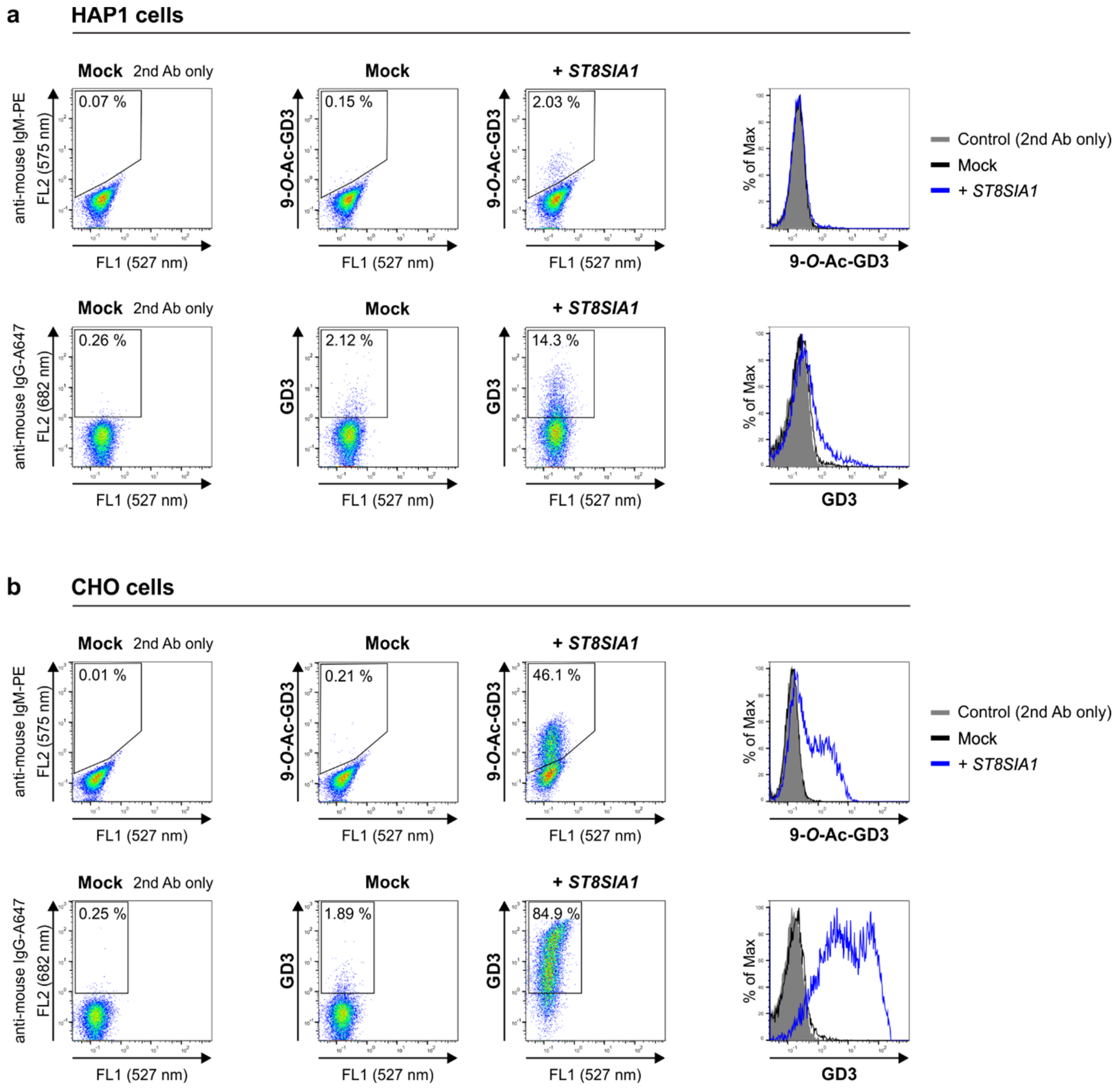

Supplementary Figure 2.

ST8SIA1-induced formation of GD3 and 9-*O*-Ac-GD3 in HAP1 and CHO cells. Flow cytometric detection of GD3 and 9-*O*-Ac-GD3 on **a,** HAP1-WT cells and **b,** CHO-WT cells. Cells were transiently transfected with human *ST8SIA1* (+ *ST8SIA1*) or empty vector (Mock). 48 hours after transfection, cells were stained with anti-9-*O*-Ac-GD3 mAb M-T6004 followed by rat anti-mouse IgM-PE (upper panel) or by anti-GD3 mAb R24 followed by goat anti-mouse IgG-A647 (lower panel). Mock transfected cells incubated with the respective secondary antibody only (2nd Ab only) were used as control (see dot plots of the far-left panel and grey fill in histograms of the far-right panel). Gates were set on positive cells and their percentages are given in the upper left corner of the dot plots. Representative data from one of two independent experiments are shown.

```
Human SLC33A1    NP_004724.1       1 MSPTISHKDSSRQRRPGNFSHSLDMKSGPLPPGGWDDSHLDSAGREGDRE     50
                                     |||||||||||||||||.|||:|||||||||||.|||||.|..|.|||||
Hamster Slc33a1  XP_003498457.1    1 MSPTISHKDSSRQRRPGMFSHALDMKSGPLPPGAWDDSHSDLVGGEGDRE     50

Human SLC33A1    NP_004724.1      51 ALLGDTGTGDFLKAPQSFRAELSSILLLLFLYVLQGIPLGLAGSIPLILQ    100
                                     |||||.|..||.|||:|:||||||||||||||||||||||||||||||||
Hamster Slc33a1  XP_003498457.1   51 ALLGDAGASDFPKAPRSYRAELSSILLLLFLYVLQGIPLGLAGSIPLILQ    100

                                              A110 S113
Human SLC33A1    NP_004724.1     101 SKNVSYTDQAFFSFVFWPFSLKLLWAPLVDAVYVKNFGRRKSWLVPTQYI    150
                                     |||||||||||||||||||||||||||||||||.||||||||||||||||
Hamster Slc33a1  XP_003498457.1  101 SKNVSYTDQAFFSFVFWPFSLKLLWAPLVDAVYFKNFGRRKSWLVPTQYI    150

Human SLC33A1    NP_004724.1     151 LGLFMIYLSTQVDRLLGNTDDRTPDVIALTVAFFLFEFLAATQDIAVDGW    200
                                     ||:||||||||||.||||||||||||:||||.||||||||||||||||||
Hamster Slc33a1  XP_003498457.1  151 LGIFMIYLSTQVDHLLGNTDDRTPDVVALTVTFFLFEFLAATQDIAVDGW    200

Human SLC33A1    NP_004724.1     201 ALTMLSRENVGYASTCNSVGQTAGYFLGNVLFLALESADFCNKYLRFQPQ    250
                                     ||||||||||||||||||||||||||||||||||||||||||||||||||
Hamster Slc33a1  XP_003498457.1  201 ALTMLSRENVGYASTCNSVGQTAGYFLGNVLFLALESADFCNKYLRFQPQ    250

Human SLC33A1    NP_004724.1     251 PRGIVTLSDFLFFWGTVFLITTTLVALLKKEN-EVSVVKEETQGITDTYK    299
                                     |||||||||||||||||||||||||||||||| |||.|||||||||||||
Hamster Slc33a1  XP_003498457.1  251 PRGIVTLSDFLFFWGTVFLITTTLVALLKKENKEVSTVKEETQGITDTYK    300

Human SLC33A1    NP_004724.1     300 LLFAIIKMPAVLTFCLLILTAKIGFSAADAVTGLKLVEEGVPKEHLALLA    349
                                     ||||||||||||.|||||||:|||||||||||||||||||||||||||||
Hamster Slc33a1  XP_003498457.1  301 LLFAIIKMPAVLAFCLLILTSKIGFSAADAVTGLKLVEEGVPKEHLALLA    350

                                                     Y366
Human SLC33A1    NP_004724.1     350 VPMVPLQIILPLIISKYTAGPQPLNTFYKAMPYRLLLGLEYALLVWWTPK    399
                                     |||||||||||||||||||||||||.||||||||||||||||||||||||
Hamster Slc33a1  XP_003498457.1  351 VPMVPLQIILPLIISKYTAGPQPLNIFYKAMPYRLLLGLEYALLVWWTPK    400
                                                     Y367

Human SLC33A1    NP_004724.1     400 VEHQGGFPIYYYIVVLLSYALHQVTVYSMYVSIMAFNAKVSDPLIGGTYM    449
                                     |||||||||||||:|||||||||||:||||||||||||||||||||||||
Hamster Slc33a1  XP_003498457.1  401 VEHQGGFPIYYYIIVLLSYALHQVTLYSMYVSIMAFNAKVSDPLIGGTYM    450

Human SLC33A1    NP_004724.1     450 TLLNTVSNLGGNWPSTVALWLVDPLTVKECVGASNQNCRTPDAVELCKKL    499
                                     |||||||||||||||||||||||||||||||||||||||||||:||||||
Hamster Slc33a1  XP_003498457.1  451 TLLNTVSNLGGNWPSTVALWLVDPLTVKECVGASNQNCRTPDAIELCKKL    500

                                              G509
Human SLC33A1    NP_004724.1     500 GGSCVTALDGYYVESIICVFIGFGWWFFLGPKFKKLQDEGSSSWKCKRNN    549
                                     ||||||||||||||||||||||||||||||||||||||||.|||||||||
Hamster Slc33a1  XP_003498457.1  501 GGSCVTALDGYYVESIICVFIGFGWWFFLGPKFKKLQDEGPSSWKCKRNN    550
                                              G510
```

Supplementary Figure 3.

Protein sequence alignment of human and hamster SLC33A1. The primary sequences of human and hamster SLC33A1 (accession no. NP_004724.1 and XP_003498457.1, respectively) were aligned by Clustal W[2]. Amino acid residues, which are affected by the patient-derived mutations analysed in this study, are conserved and highlighted by using the following colour code: HBS-derived variants A110P and Y366*, blue; SPG42-derived variant S113R, red; ATX-derived G509S, yellow. Y366 and G509 in human SLC33A1 correspond to Y367 and G510 in hamster SLC33A1.

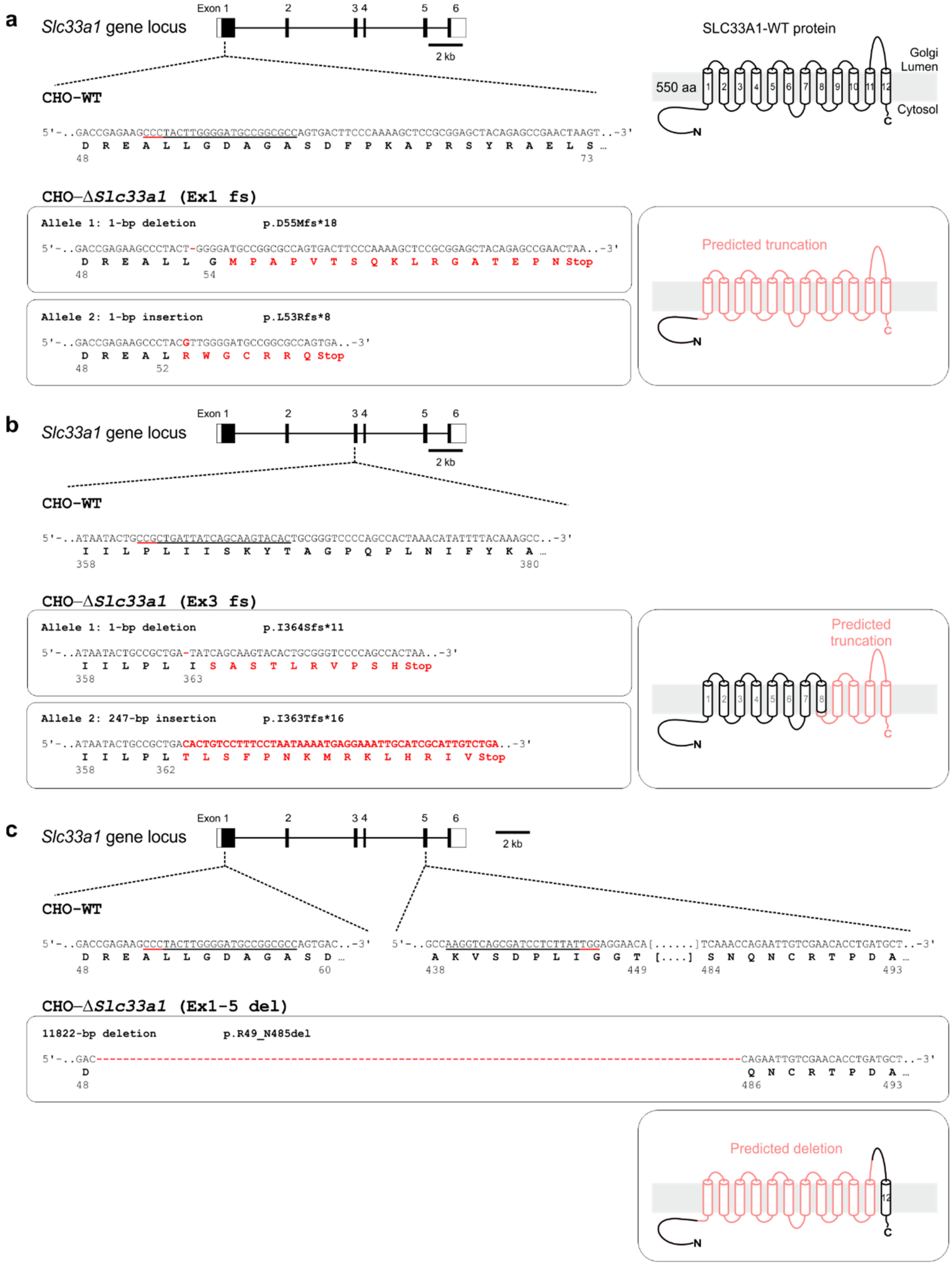

Supplementary Figure 4.

Genetic alterations in newly generated CHO-Δ*Slc33a1* clones. Schematic overview of the *Slc33a1* gene locus of CHO-WT cells showing the target sites used for genetic inactivation of *Slc33a1* by CRISPR/Cas9-mediated genome editing. **a,** Single-gRNA approach resulting in CHO-Δ*Slc33a1* (Ex1 fs); **b,** Single-gRNA approach resulting in CHO-Δ*Slc33a1* (Ex3 fs); **c,** Two-gRNA approach resulting in CHO-Δ*Slc33a1* (Ex1-5 del). The target sequence of each gRNA is underlined with the PAM site underlined in red. Sequence alterations in the selected Δ*Slc33a1* clones are highlighted in red. A scheme showing the topology model of SLC33A1 is provided with the predicted protein truncation/deletion shown in red. Genetic alterations have been validated on genomic and transcript level by PCR and RT-PCR, respectively, followed by Sanger sequencing of the obtained PCR products.

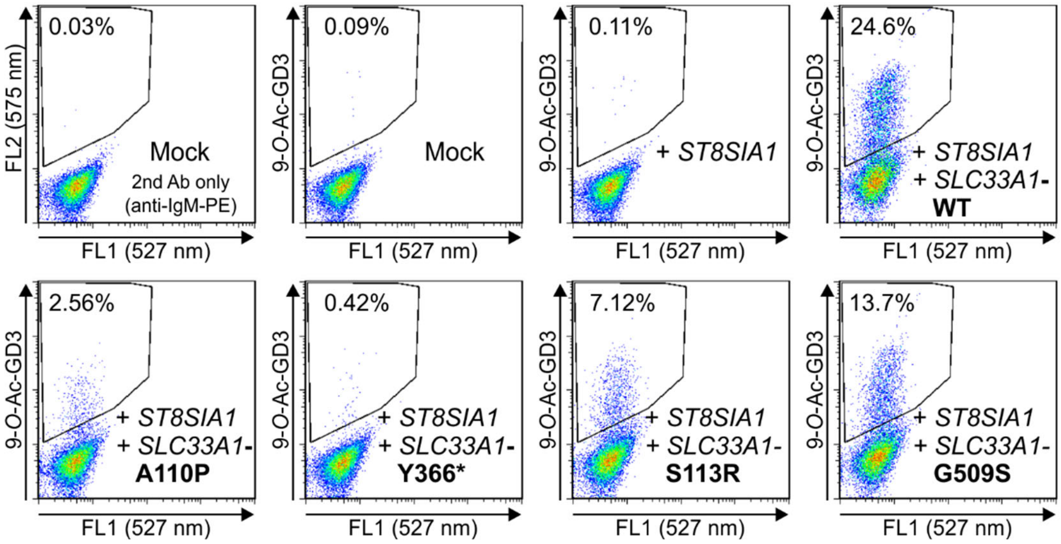

Supplementary Figure 5.

Gating of 9-*O*-Ac-GD3-positive CHO-Δ*Slc33a1* cells obtained after complementation with human *SLC33A1* variants. CHO-Δ*Slc33a1* (Ex1-5 del) cells were transiently transfected with a plasmid encoding human V5-tagged ST8SIA1 (+ *ST8SIA1*), alone or in combination with a plasmid encoding one of the indicated human SLC33A1-variants. Cells transfected with empty vector (Mock) were used as controls. Cells were stained with anti-9-*O*-Ac-GD3 mAb M-T6004 followed by anti-mouse IgM-PE and flow cytometric analysis. Mock-transfected cells stained with secondary antibody only (2nd Ab only) served as additional control. Representative dot plots from one of four independent experiments are shown exemplarily to illustrate the gating strategy used for the quantification of 9-*O*-Ac-GD3-positive cells. Their percentages are given in the upper left corner of the dot plot. The respective numbers from all four experiments are summarized in Fig. 3b.

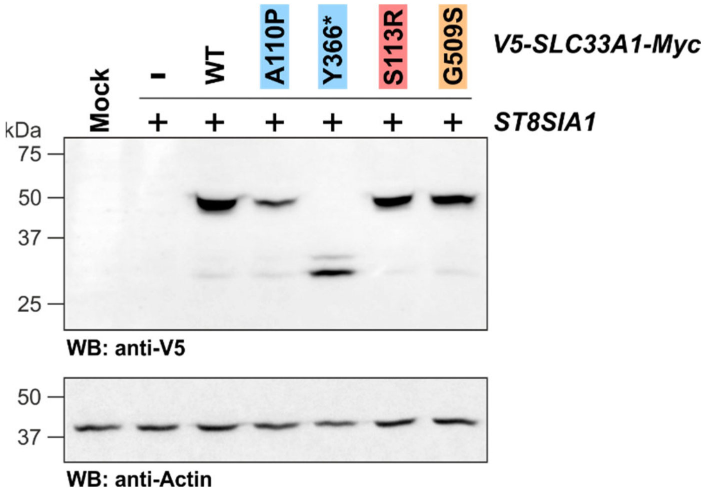

Supplementary Figure 6.

Western Blot analysis of V5-tagged SLC33A1 variants. CHO-Δ*Slc33a1* (Ex1-5 del) cells were transiently transfected with a plasmid encoding human Flag-tagged ST8SIA1, alone or in combination with a plasmid encoding the indicated N-terminally V5-tagged SLC33A1 variants. Mock transfected cells were used as negative control and Actin as loading control. This experiment replicates the approach shown in Fig. 3b, except that the epitope tags on the expressed proteins have been switched. Instead of using Flag-tagged SLC33A1 and V5-tagged ST8SIA1, we here employed V5-tagged SLC33A1 and Flag-tagged ST8SIA1. All other conditions were kept consistent with the original experimental design. Representative blot from two independent experiments is shown.

In the current setup, immuno-positive bands are present in all lysates of *V5-SLC33A1*-transfected cells but absent from those of the two negative controls (mock and *ST8SIA1*-only transfected cells). The C-terminally truncated variant SLC33A1-Y366* migrated as a doublet of about 35 and 30 kDa, of which the lower band may correspond to a more stable degradation product. All full-length variants of SLC33A1 migrated as a prominent band of about 50 kDa, accompanied by a faint band of about 30 kDa that may represent the same degradation product as seen for SLC33A1-Y366*.

Source data are provided as a Source Data file.

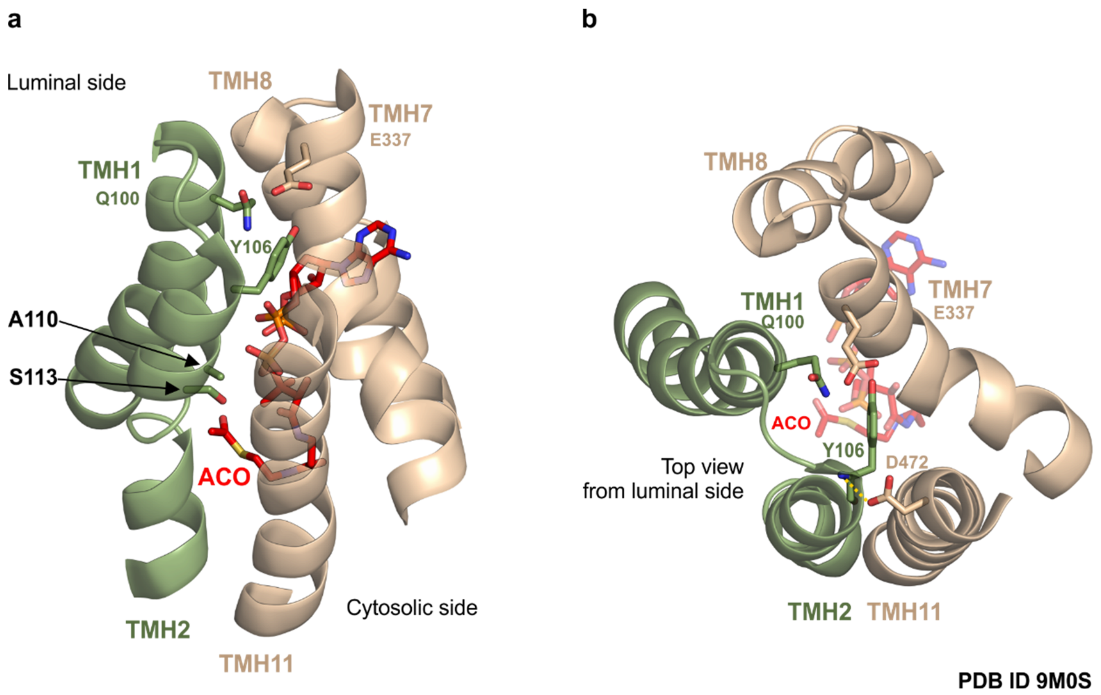

Supplementary Figure 7.

Positioning of Y106 in the inward-facing conformation of SLC33A1. Detail of the cryo-EM strucutre of SLC33A1 (PDB ID 9M0S)[1], showing the luminal parts of TMH1-2, TMH7-8 and TMH11. TMHs that belong to the N-terminal and C-terminal six-helix bundel of SLC33A1 are shown in green and beige, respectively. Selected amino acid side chains are shown in stick representation. Bound acetyl-CoA (ACO, red) is shown stick representation. **a,** Oblique view. The location of TMH2 residues that are mutated in the patient-derived variants p.A110P and p.S113R are indicated by black arrows. The ribbon representation is rendered as partially transparent to facilitate visualization of the bound acetyl-CoA. **b,** Top view from luminal side. H-bonding between the main chain of Y106 and the side chain of D472 is indicated by yellow dots. Ribbon representation drawings were prepared in PyMol.

The cryo-EM structure of SLC33A1 solved by Zhou et al. captured an in the inward-facing conformation of SLC33A1, in which the luminal face of the acetyl-CoA binding cavity is occluded by TMH1-2 and TMH7-8, stabilized by lateral closure of TMH2 and TMH11[1]. Closer inspection of the cryo-EM structure revealed that the side chains of Q100, Y106 and E337 contribute to the sealing of the luminal face. Moreover, the main chain amide of Y106 is engaged in interhelical main chain-side chain H-bonding with D472, thereby holding the luminal tips of TMH2 and TMH11 together for lateral closure.

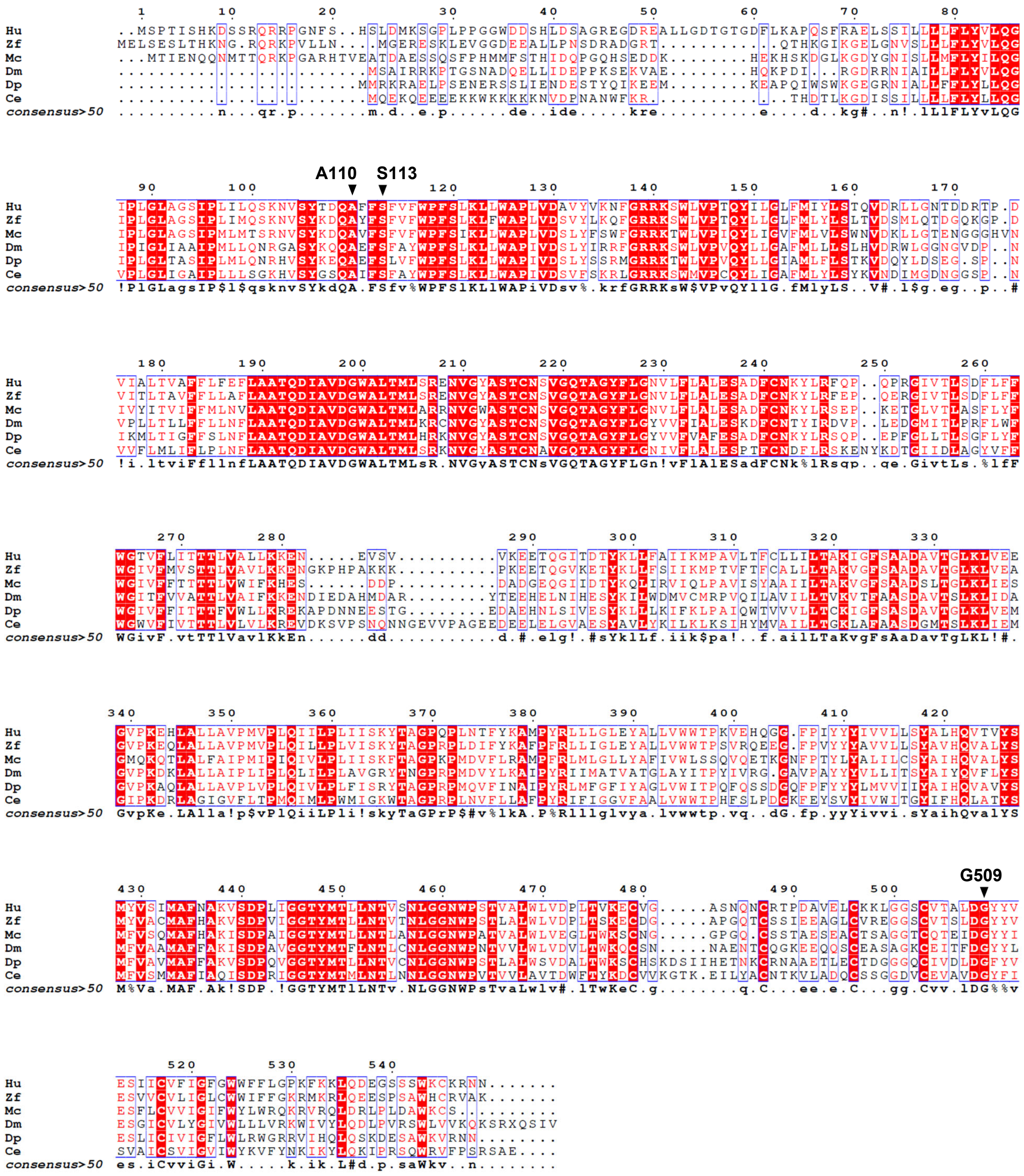

Supplementary Figure 8.

The human SLC33A1 residues A110, S113 and G509 are highly conserved across SLC33A1 orthologs of vertebrate and invertebrate species. Multiple sequence alignment of human (Hu) SLC33A1 with exemplarily selected protein orthologs from Chordata/Teleostei (*Danio rerio* - zebrafish, Zf), Nematoda (*Caenorhabditis elegans*, Ce), Mollusca (*Mytilus californianus*, Mc), Arthropoda/Hexapoda (*Drosophila melanogaster*, Dm) and Arthropoda/Crustacea (*Daphnia pulex*, Dp). The numbering on top of the alignment blocks refers to the amino acid numbering of human SLC33A1. The consensus sequence shows the residue types most prevalent (>50%) at each position. Residues highlighted by red boxes show 100% conversation. The human SLC33A1 residues A110, S113 and G509, for which the

substitutions A110P, S113R and G509S were found in patients with Huppke-Brendel syndrome, Spastic paraplegia 42 and late-onset cerebellar ataxia, respectively, are indicated by black arrow heads.

Multiple sequence alignment was performed using MultAlin[3]; the graphical output was generated by ESPrit 3.0 (https://espript.ibcp.fr/ESPript/ESPript/)[4]. Accession numbers of the used SLC33A1 sequences are as follows: NP_001177921.1 (human), NP_957402.2 (*Danio rerio*), NP_001356982.1 (*Drosophila melanogaster*), XP_046446276.1 (*Daphnia pulex*), NP_495970.1 (*Caenorhabditis elegans*), XP_052102896.1 (*Mytilus californianus*).

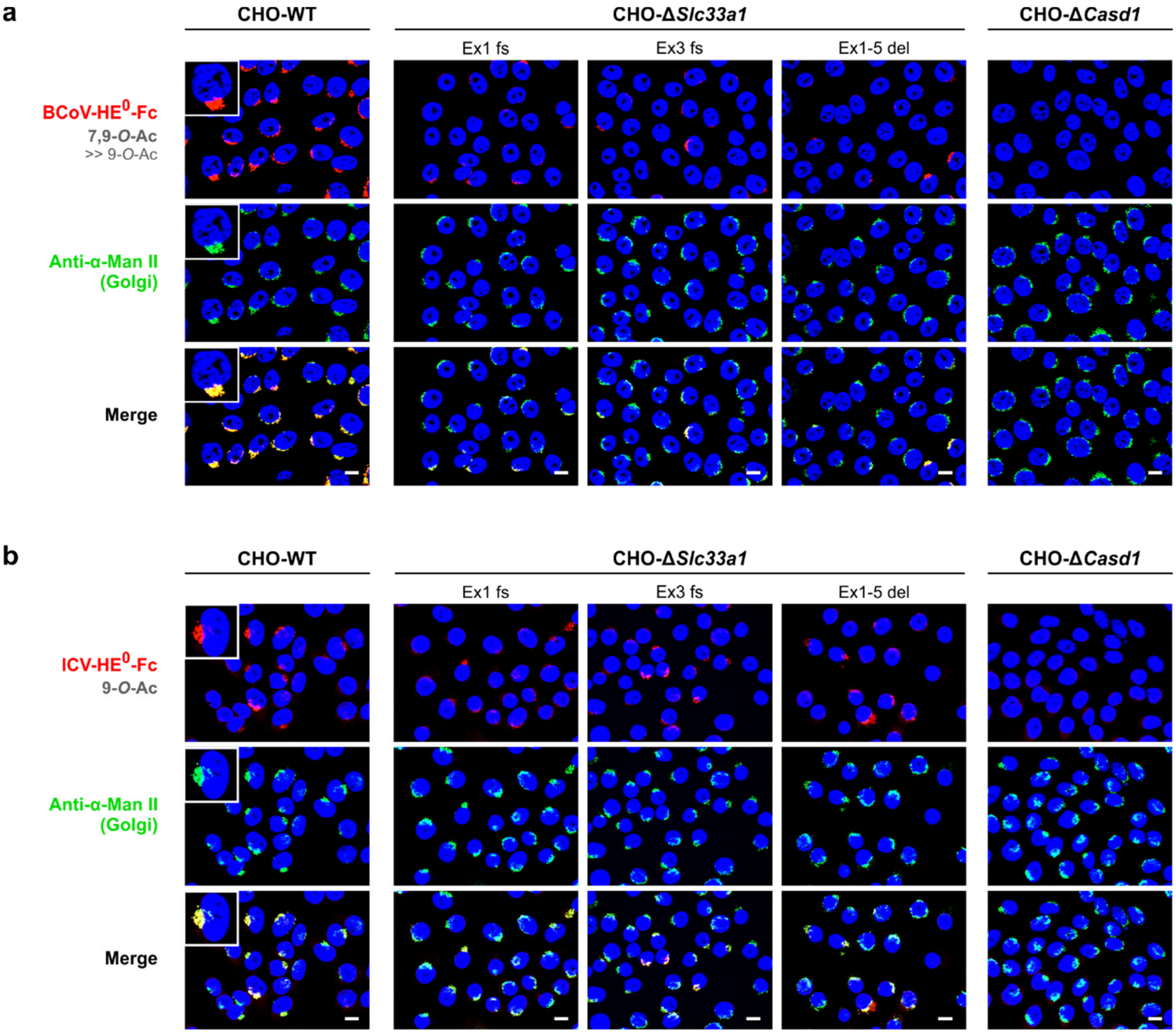

Supplementary Figure 9.

Golgi localization of intracellular virolectin ligands. Fluorescence microscopy images of Triton-permeabilized CHO cells co-stained with virolectin (red) and an antibody that recognizes the Golgi marker α-mannosidase II (anti-α-Man II, green). Colocalized red and green signals appear in yellow (merge). Nuclei were counterstained with DAPI (blue). **a.** Staining with the virolectin BCoV-HE^0-Fc for the detection of 7,9-*O*-acetylated sialoglycans. Scale bars: 10 µm. **b.** Staining with the virolectin ICV-HE^0-Fc for the detection of 9-*O*-acetylated sialoglycans. Scale bars: 10 µm. Insets show an enlarged image of a representative CHO-WT cell to illustrate Golgi localization of 7,9-*O*- and 9-*O*-acetylated sialosides. Representative images from one of two independent experiments are shown. DAPI, 4′,6-diamidino-2-phenylindole.

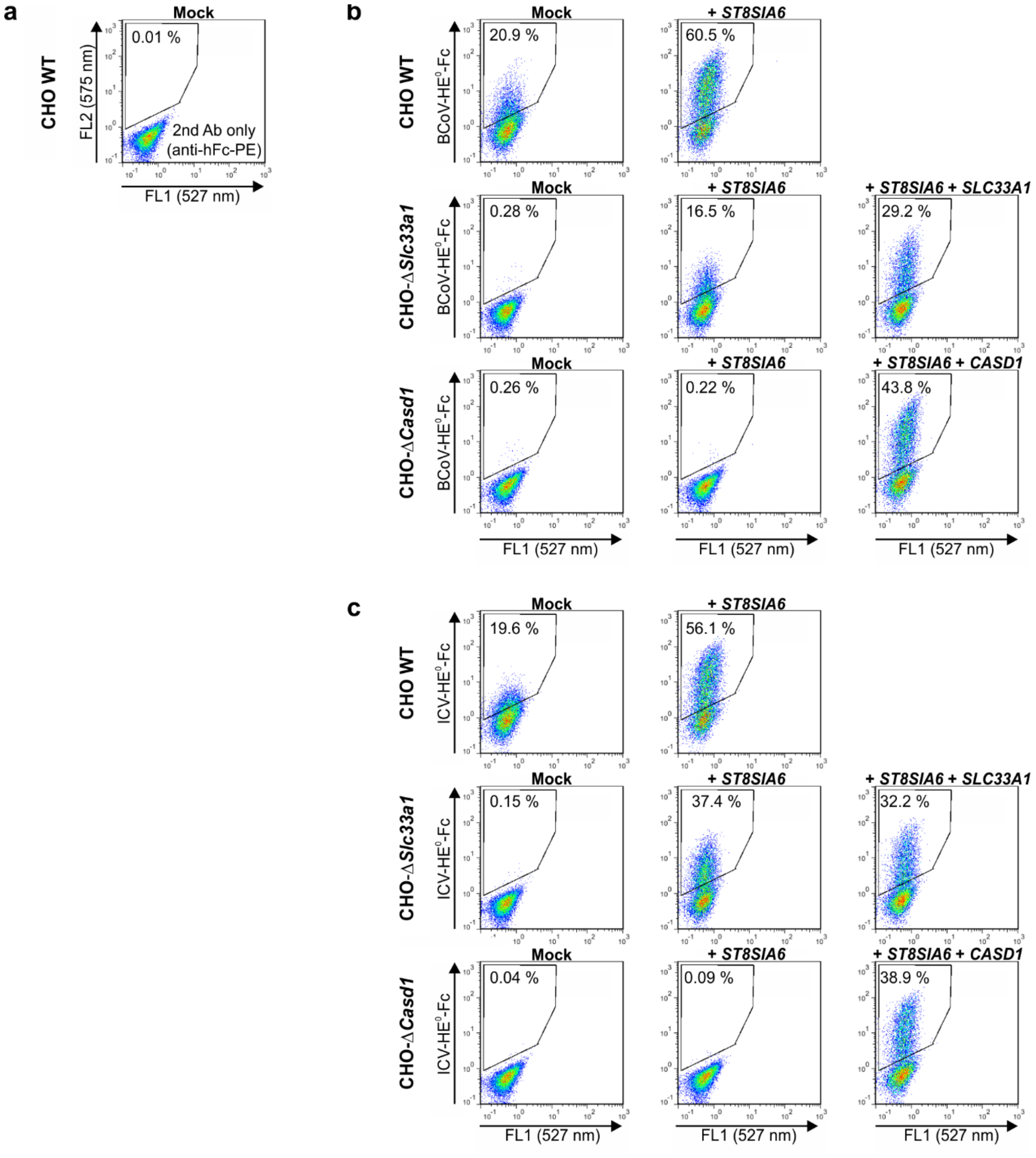

Supplementary Figure 10.

Gating strategy used for the flow cytometric analyses shown in Figure 4. a, Mock-transfected CHO-WT cells incubated with secondary antibody only; **b**, CHO cells of the indicated genotype stained with the virolectin BCoV-HE^{0}-Fc (detection of 7,9-*O*-acetylated sialosides); **c**, CHO cells of the indicated genotype stained with the virolectin ICV-HE^{0}-Fc (detection of 9-*O*-acetylated sialosides). Prior to analysis, CHO-WT, -Δ*Slc33a1* and -Δ*Casd1* cells were transiently transfected with a *V5-ST8SIA6* construct, either alone (+ *ST8SIA6*) or in combination with *Flag-SLC33A1-Myc* (+ *ST8SIA6* + *SLC33A1*) or *Myc-CASD1* (+ *ST8SIA6* + *CASD1*). Mock transfected cells were used as controls. Transfected cells were stained with virolectin followed by PE-conjugated anti-human Fc pAb. Representative dot plots from one of four independent transfection sets are shown, illustrating the gating strategy used for the quantification of positive cells. Data from all four transfection sets are summarized in Fig. 4d for CHO-WT cells, in Fig. 4f for CHO-Δ*Slc33a1* cells and in Fig. 4h for CHO-Δ*Casd1* cells.

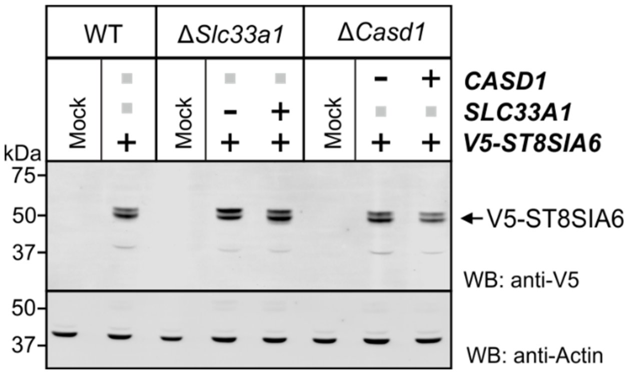

Supplementary Figure 11.

Control experiment demonstrating that *Slc33a1*-deficiency does not affect *ST8SIA6* expression. Representative Western blot analysis of cells from one of the four transfection sets which were analysed by flow cytometry in Fig. 4d (CHO-WT), Fig. 4f (CHO-Δ*Slc33a1*) and Fig. 4h (CHO-Δ*Casd1*). CHO cells of the indicated genotype were transfected with *V5-ST8SIA6* with (+) and without (-) co-transfection of *Flag-SLC33A1-Myc* or *Myc-CASD1*. Cells transfected with empty vector (Mock) were used as negative controls. The presence of endogenous *Casd1* and/or *Slc33a1* in each genotype is indicated by grey squares. Total lysates of transfected cells were separated by 10% SDS-PAGE, followed by immunoblotting with anti-V5 mAb (upper panel). An arrow indicates the migration position of glycosylated V5-tagged ST8SIA6. Immunodetection of actin was used as a loading control (bottom panel). WB, Western Blot. Representative blots from one of three independent experiments are shown.

Source data are provided as a Source Data file.

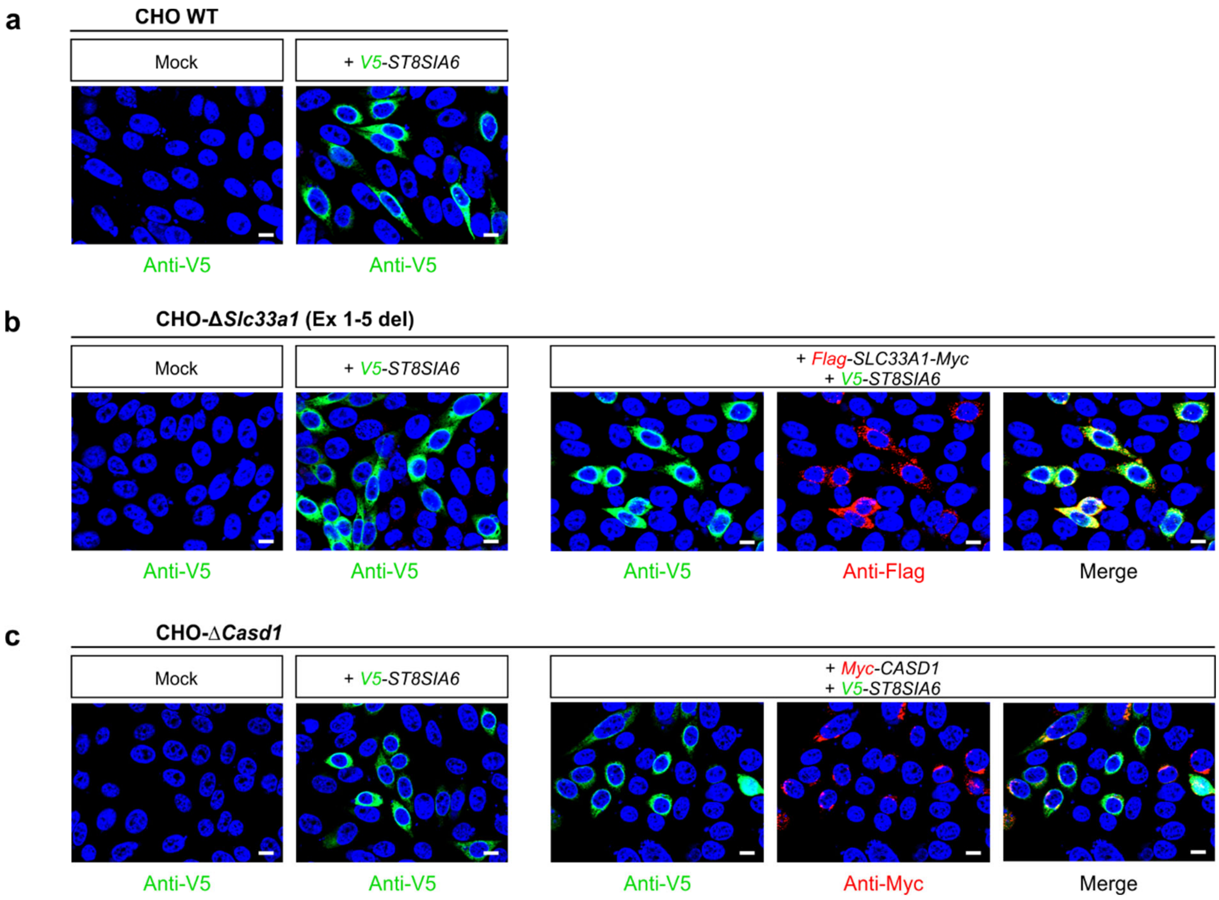

Supplementary Figure 12.

Control experiment validating the expression of V5-ST8SIA6, Flag-SLC33A1-Myc and Myc-CASD1 in CHO cells. Fluorescence microscopy images of **a**, permeabilized CHO-WT cells stained with anti-V5 mAb (green); **b**, permeabilized CHO-Δ*Slc33a1* (Ex 1-5 del) stained with anti-V5 mAb (green) and anti-Flag pAb (red); and **c**, permeabilized CHO-Δ*Casd1* cells stained with anti-V5 pAb (green) and anti-Myc mAb (red). Prior to staining, cells were transiently transfected with *V5-ST8SIA6* alone or in combination with *Flag-SLC33A1-Myc* or *Myc-CASD1* as described in the legend of Fig. 4b,e,g. Transfected cells were fixed and permeabilized with Triton X-100. Mock transfected cells were used as controls. Cell nuclei were counterstained with DAPI (blue). Scale bars: 10 µm. Representative images from one of three independent experiments are shown. DAPI, 4′,6-diamidino-2-phenylindole.

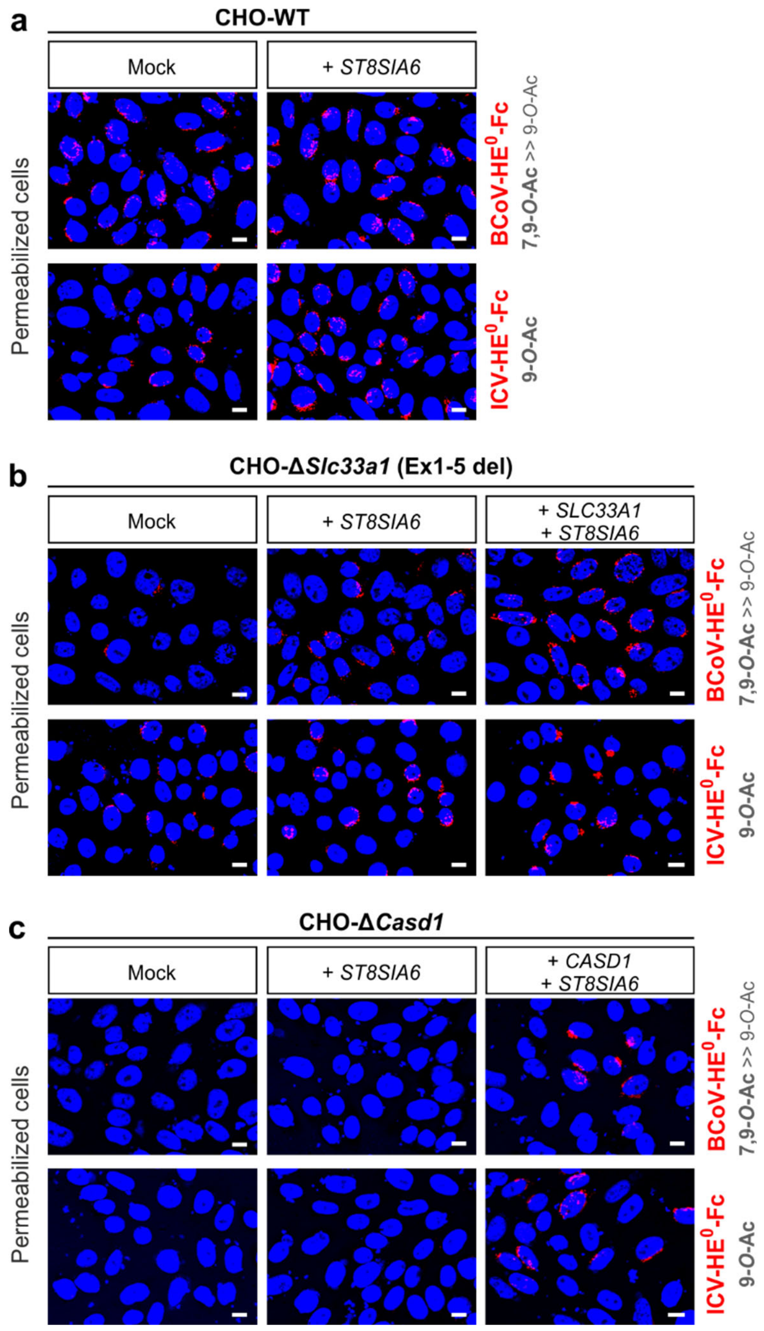

Supplementary Figure 13.

Intracellular virolectin ligands in CHO-WT and mutant cells transfected as in Fig. 4b,e,g. Fluorescence microscopy images of Triton-permeabilized CHO-WT (**a**), CHO-Δ*Slc33a1* (**b**) and CHO-Δ*Casd1* cells (**c**) stained with the indicated virolectins (red). Nuclei were counterstained with DAPI (blue). Scale bars: 10 µm. The data correspond to the data sets shown in Fig. 4b,e,g for non-permeabilized cells. Please note that intracellular virolectin ligands that are present before *ST8SIA6* expression (mock) contribute to the total staining signal detected after *ST8SIA6* expression (+ *ST8SIA6*). Representative images from one of three experiments are shown. DAPI, 4′,6-diamidino-2-phenylindole.

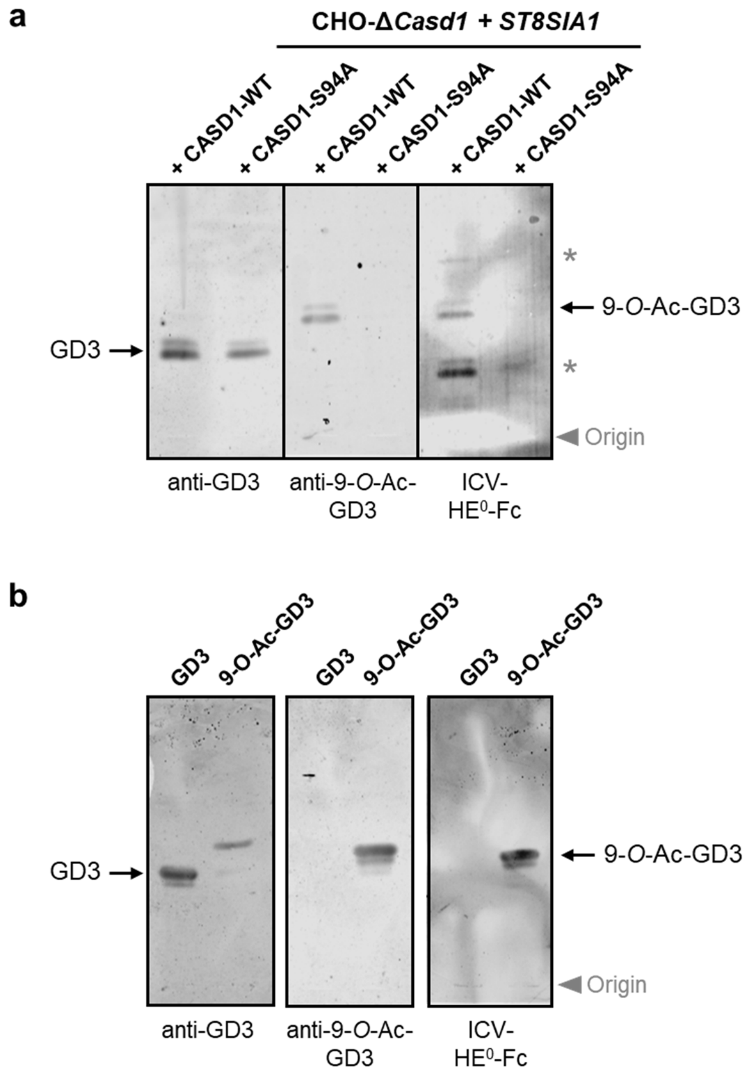

Supplementary Figure 14.

Virolectin staining confirms essential role of CASD1's luminal catalytic domain in 9-*O*-acetyl-GD3 synthesis. a, Immuno-TLC analysis of total gangliosides from CHO-Δ*Casd1* cells transiently co-transfected with plasmids encoding ST8SIA1 and either CASD1-WT or the mutant CASD1-S94A, in which the luminal catalytic domain (LCD) is specifically inactivated while the C-terminal transmembrane region (CTMR) remains intact. Gangliosides were separated by thin-layer chromatography (TLC) and probed with anti-GD3 monoclonal antibody (mAb) R24, anti-9-*O*-Ac-GD3 mAb UM4D4 or the virolectin ICV-HE^0-Fc. Loss of the 9-*O*-acetyl-GD3 signal in CASD1-S94A expressing cells—detected by both UM4D4 and ICV-HE^0-Fc—confirms that the LCD of CASD1 is essential for the ST8SIA1-mediated formation of 9-*O*-Ac-GD3. Notably, ICV-HE^0-Fc also recognized additional 9-*O*-acetylated ganglioside species (asterisks), indicating broader reactivity. The identities of these ganglioside species and the potential contribution of other sialyltransferases to their biosynthesis remain to be characterized. **b,** Specificity validation of detection reagents using defined ganglioside standards. GD3 and 9-*O*-acetyl-GD3 were resolved by TLC and stained with R24, UM4D4, or ICV-HE^0-Fc. Both UM4D4 and ICV-HE^0-Fc specifically recognized 9-*O*-acetyl-GD3 but not the non-*O*-acetylated GD3, confirming the specificity of the detection system.

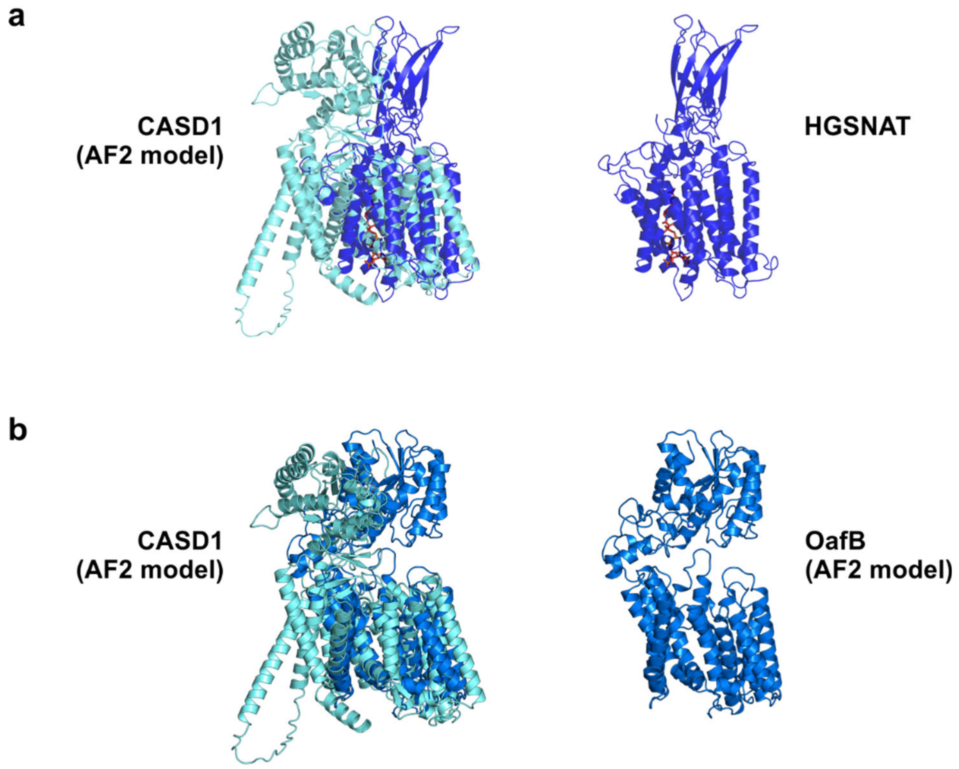

Supplementary Figure 15.

Structural comparison of CASD1 with HGSNAT and OafB. a, Superposition of the AF2 model of human CASD1 (cyan) with the cryo-EM structure of human HGSNAT (blue) solved in the acetyl-CoA (red) bound state (PDB 8TU9)[5]. **b**, Superposition of the AF2 model of CASD1 (cyan) and the AF2 model of OafB (aquamarine) from *Salmonella typhimurium*. Structural homology between all three proteins is restricted to the 9-TMH core as shown in Fig. 6b. The SGNH domains of CASD1 and OafB are structurally related, but are predicted to have a different orientation with respect to the membrane.

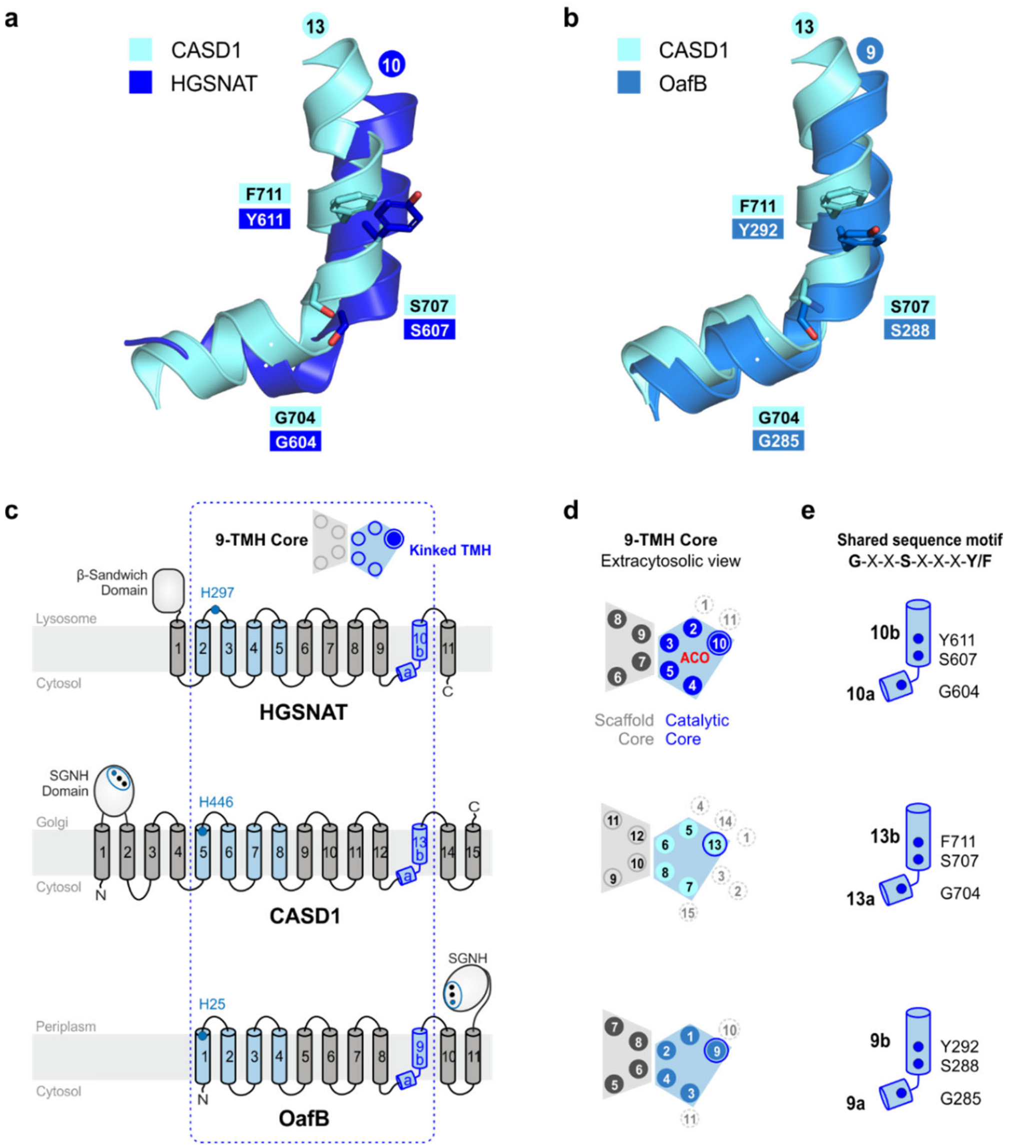

Supplementary Figure 16.

The characteristic kink in the C-terminal TMH of the catalytic core of HGSNAT (TMH10) is conserved in CASD1 and OafB. The cryo-EM structure of HGSNAT revealed splitting of the last 'catalytic core'-TMH into two kinked half-helices (TMH10a/b) with a cross-angle of around 90° [5, 6, 7]. This characteristic feature of HGSNAT appears to be conserved in CASD1 and OafB. **a,b.** Enlarged detail of the superposition of (**a**) the AF2 model of CASD1 (cyan) with the cryo-EM structure of HGSNAT (PDB ID 8TU9[5]; blue) and (**b**) the AF2 models of CASD1 (cyan) and OafB (aquamarine). Cartoon representation of the last 'catalytic core'-TMH (TMH10, TMH13 and TMH9 in HGSNAT, CASD1, and Oafb, respectively). Positional conserved residues of a shared **G**-X-X-**S**-X-X-X-**Y**/**F** motif are shown in stick representation. TMH numbers are indicated on top. **c,d**. Modified versions of the schemes shown in Fig. 6a,b with the kinked TMH highlighted by blue rendering. **e**. Schematic representation of the kinked TMH with the residues of the **G**-X-X-**S**-X-X-X-**Y**/**F** motif indicated as blue dots.

The amino acid numbering for human HGSNAT used throughout this manuscript is based on the canonical UniProt sequence Q68CP4. In Q68CP4, the position of the catalytic histidine is H297[6, 7], which corresponds to H269[5] in the numbering system that sets the second methionine (M29 in Q68CP4) as amino acid 1.

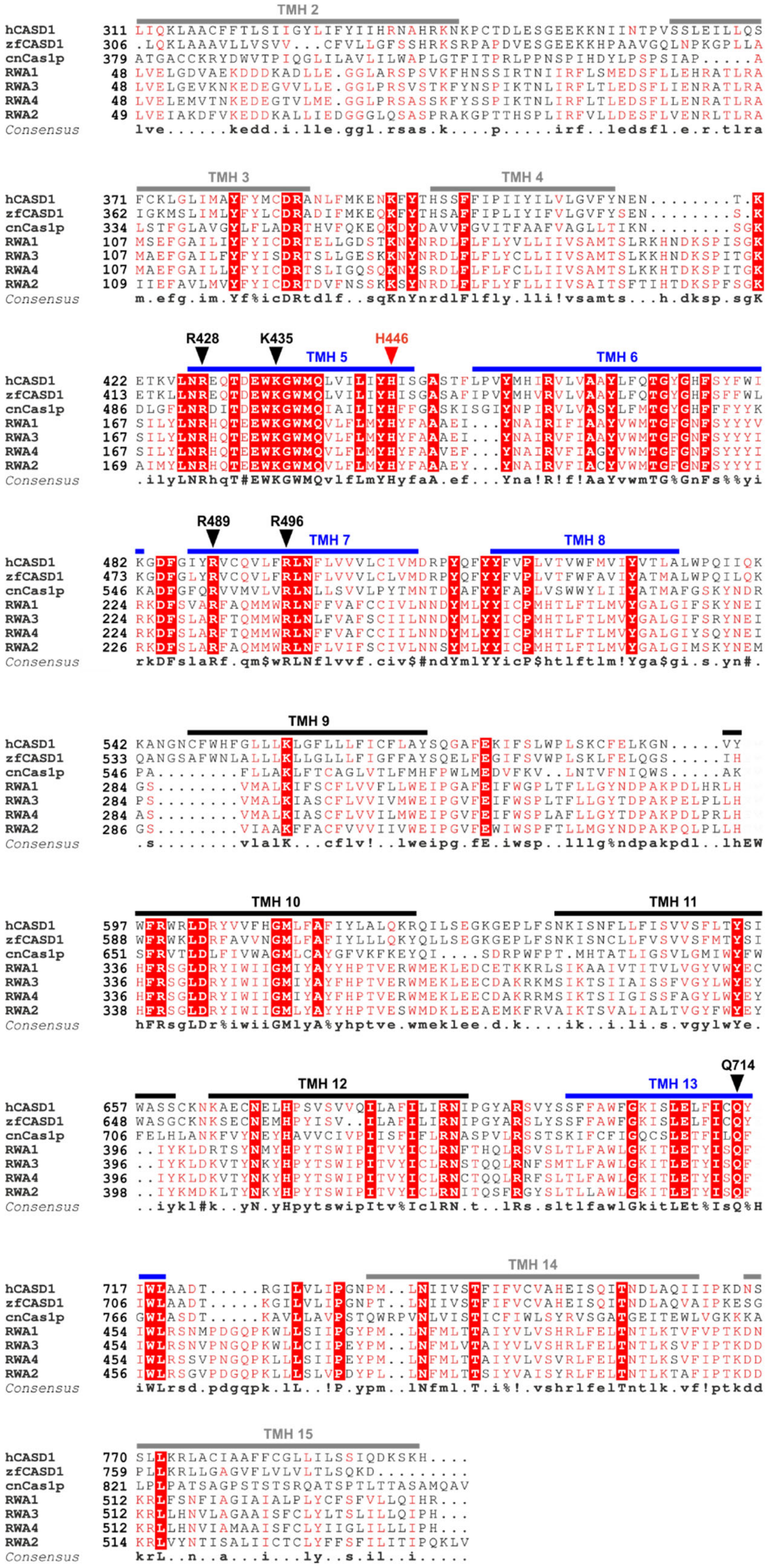

Supplementary Figure 17.

Functional relevant residues identified in the CTMR of human CASD1 are conserved among orthologous variants of the CTMR from zebrafish, Arabidopsis and the encapsulated basidiomycetous yeast *Cryptococcus neoformans*. Multiple sequence alignment of the CTMR of

human CASD1 (hCASD1) with corresponding sequence stretches of orthologues proteins from zebrafish (zfCASD1), *Cryptococcus neoformans* (cnCas1p) and *Arabidopsis thaliana* (RWA1 to RWA4). The numbers on the left of each line give the position of the first amino acid residue in the respective sequences. The consensus sequence provided below each alignment block shows the residue types most prevalent (>50%) at each position. Residues highlighted by red boxes show 100% conversation. The positions of the C-terminal TMHs of human CASD1 as deduced from the corresponding AF2 model are provided above the aligned sequences. TMHs of the 9-TMH core are depicted in blue (catalytic core) and black (scaffold core). Additional TMHs are shown in grey.

The position of the conserved residue H446 (TMH 5), which has been identified in this study as the active-site residue of the CTMR of human CASD1, is indicated by a red arrow head. The position of other critical residues identified in this study are indicated by a black arrow head.

Multiple sequence alignment was performed using MultAlin[3]; the graphical output was generated by ESPrit 3.0 (https://espript.ibcp.fr/ESPript/ESPript/)[4] followed by manual curation. Uniprot accession numbers of the used sequences are as follows: hCASD1 Q96PB1 (797 aa; residues 311-797), zfCASD1 Q1LW89 (781 aa; residues 306-781), cnCAS1 Q8X226 (959 aa; residues 379-852), RWA1 Q8L7C8 (540 aa; residues 48-540), RWA2 Q0WW17 (545 aa; residues 49-545), RWA3 Q66GQ5 (540 aa; residues 48-540), and RWA4 Q9FXG3 (540 aa; residues 48-540). Numbers given in brackets indicate the total number of amino acids (aa) and the actual sequence stretch that has been used as input for the alignment program.

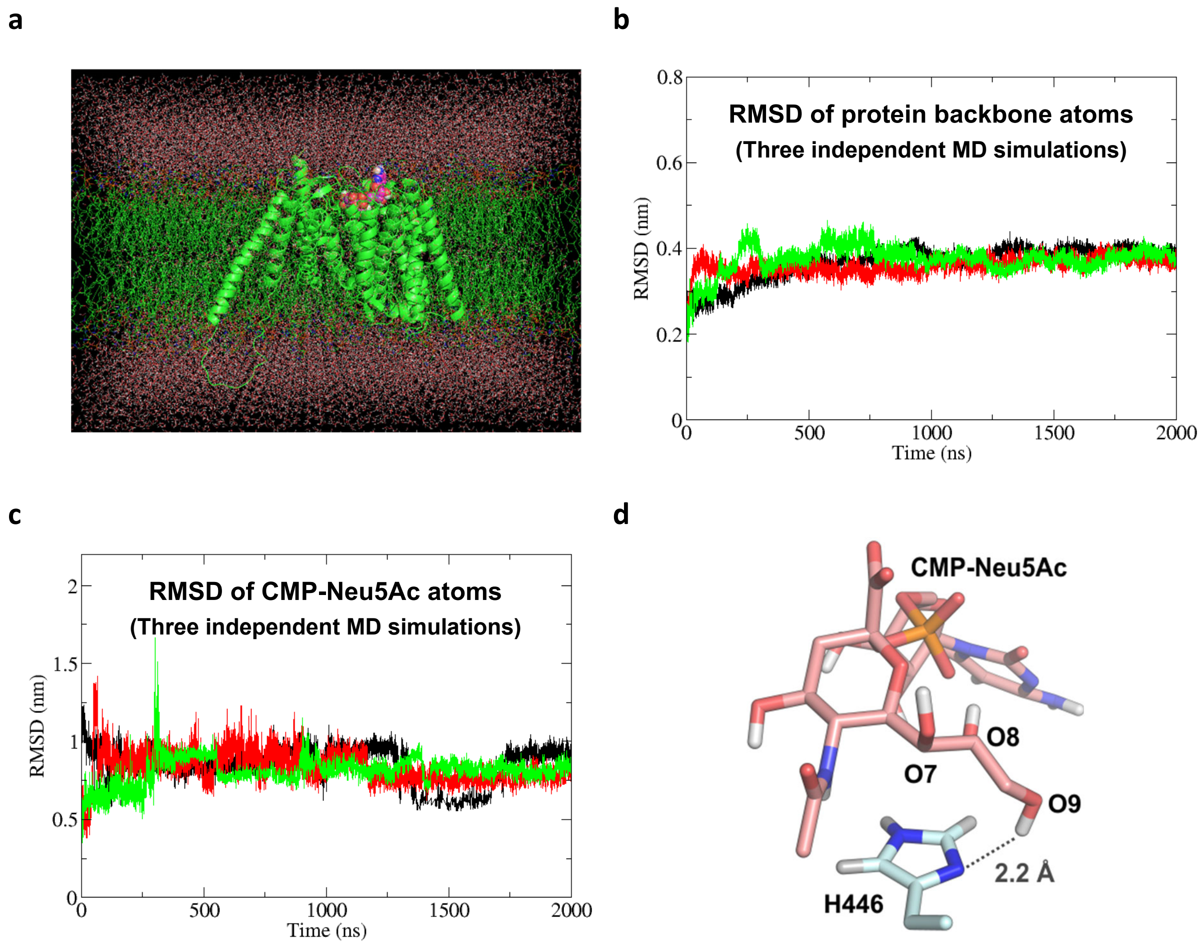

Supplementary Figure 18.

Molecular dynamics investigation of the acceptor-binding site of the C-terminal transmembrane region (CTMR) of CASD1. a, Initial CTMR–CMP-Neu5Ac complex embedded in a 1,2-dipalmitoyl-sn-*glycero*-3-phosphocholine (DPPC; dark green lines) lipid bilayer model used for molecular dynamics (MD) simulation. The CTMR secondary structural elements of CASD1 are displayed in light green. CMP-Neu5Ac is displayed in coloured spheres, and water molecules are shown as red lines. **b** and **c,** Protein (**b**) and ligand (**c**) root-mean-square deviation (RMSD) plot of three independent MD simulations, that commenced from different initial velocity distribution (black and red) and a different configuration (green). The protein structure (**b**) took 200-250 ns to equilibrate in the model lipid bilayer and remained stable for the rest of the MD simulation. Notably, in two of the runs (**c**, green and red) the CMP-Neu5Ac ligand rapidly reoriented in the binding pocket and remained stable throughout the last 1000 ns of MD simulations, while in the third simulation run (**c**, black) the CMP-Neu5Ac ligand showed larger fluctuations. The relative fluctuations in CMP-Neu5Ac are primarily due to the molecule's flexibility and importantly all three simulations converge to a similar binding state within 2000 ns. **d,** Detail of the converged structure snapshot after 2000 ns MD simulation. H446 and CMP-Neu5Ac are shown in stick representation with carbon atoms coloured in cyan and salmon, respectively. The dotted line represents the H-bonding distance between Nδ1 of H446 and the acceptor 9'-hydroxyl group of CMP-Neu5Ac.

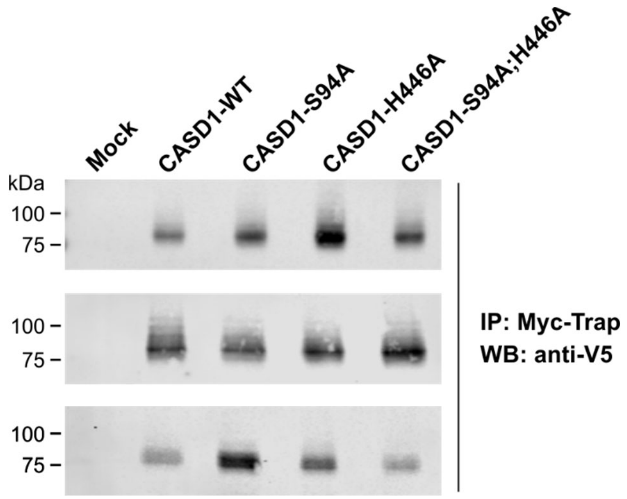

Supplementary Figure 19.

Loading controls for the on-bead assay shown in Fig. 6f. WT and mutant forms of N-terminally Myc-V5-tagged CASD1 were expressed in CHO-Δ*Casd1* cells and were affinity captured from the detergent lysate of 1.5 × 10^7 cells with an anti-Myc nanobody-coupled to magnetic agarose beads (Myc-Trap). Enzyme-loaded beads were used in the on-bead assay shown in Fig. 6f. After the reaction, beads were collected by magnetic separation and the enzyme loading was validated by Western blotting using an anti-V5 antibody. Blots from three biological replicates are shown, corresponding to the data set in Fig. 6f. IP, immunoprecipitation; WB, Western blot.

Source data are provided as a Source Data file.

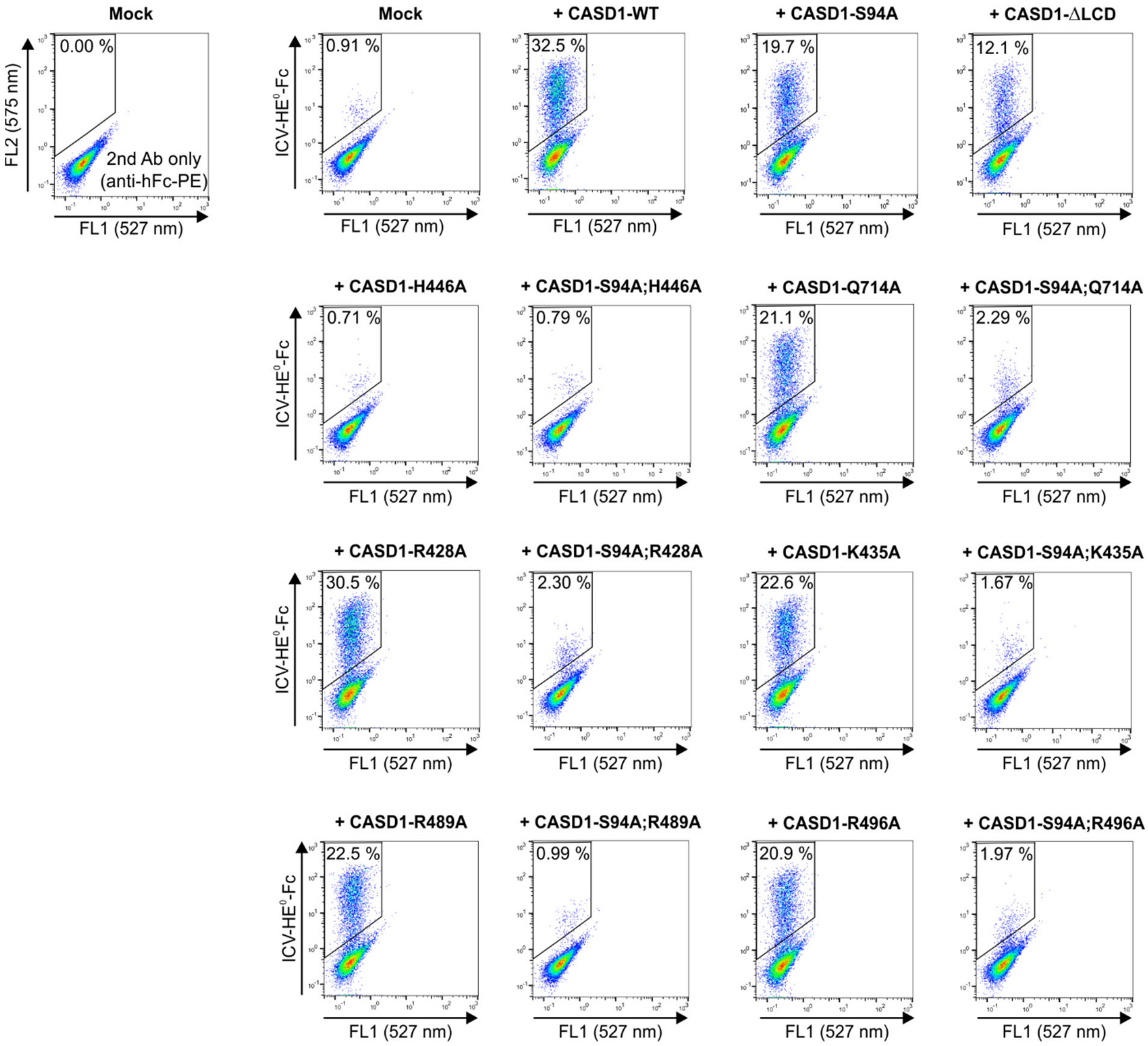

Supplementary Figure 20.

Gating strategy for the virolectin-based flow cytometry analysis of CHO-Δ*Casd1* cells shown in Figure 6g. CHO-Δ*Casd1* cells were co-transfected with a plasmid encoding human ST8SIA6 along with either empty vector (Mock) or a plasmid encoding one of the indicated CASD1 variants. Cells were stained with the virolectin ICV-HE^0-Fc followed by anti-human Fc-PE. Representative dot plots from one of three independent experiments are shown, illustrating the gating strategy used to determine the percentage of positive cells. Data from all three experiments are summarised in Fig. 6g.

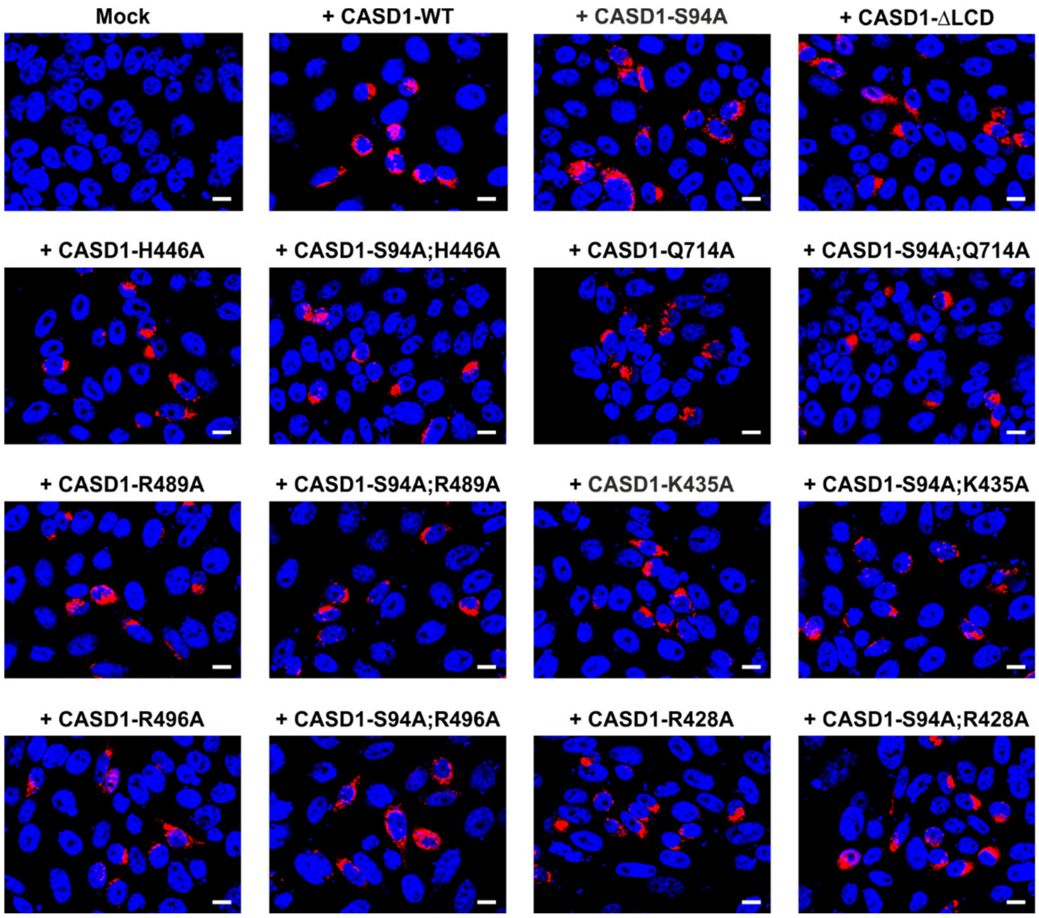

Supplementary Figure 21.

Control experiment validating the expression of the CASD1 variants analyzed in Fig. 6g. Fluorescence microscopy images of permeabilized CHO-Δ*Casd1* cells stained with anti-Myc mAb followed by donkey anti-mouse IgG Alexa 555 (red); Prior to staining, cells were transiently transfected with empty vector (mock) or a plasmid encoding one of the indicated Myc-V5-tagged CASD1 variants, as described in the legend of Fig. 6g. Transfected cells were fixed and permeabilized with Triton X-100. Cell nuclei were counterstained with DAPI (blue). Scale bars: 10 µm. Representative images from one of three independent experiments are shown. DAPI, 4′,6-diamidino-2-phenylindole.

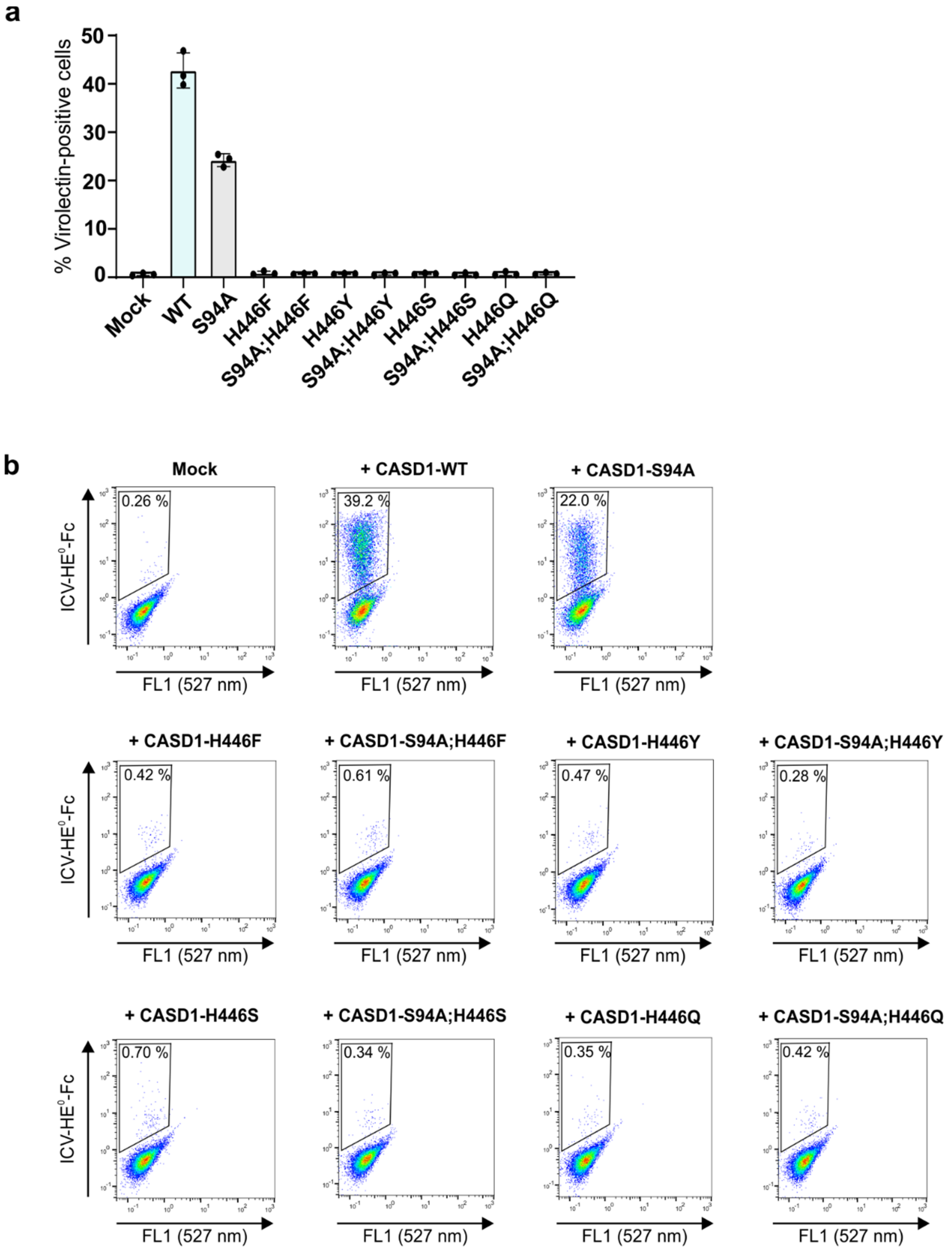

Supplementary Figure 22.

Complementation activity of CASD1 variants carrying different substitutions at position H446. CHO-Δ*Casd1* cells were transiently transfected with *ST8SIA6* along with either empty vector (mock), CASD1-WT (WT) or one of the indicated CASD1 mutants. Cells were analyzed 24 h after transfection by flow cytometry using the virolectin ICV-HE^0-Fc followed by staining with an anti-Fc-PE secondary antibody. **a,** Bar graph summarizing the quantification of three biological replicates. WT and mutant forms of CASD1 are represented by blue and grey bars, respectively. The percentage of virolectin-positive cells is given as mean ± SD (n = 3). **b,** Representative dot plots from one of three independent experiments are shown, illustrating the gating strategy to determine the percentage of positive cells.

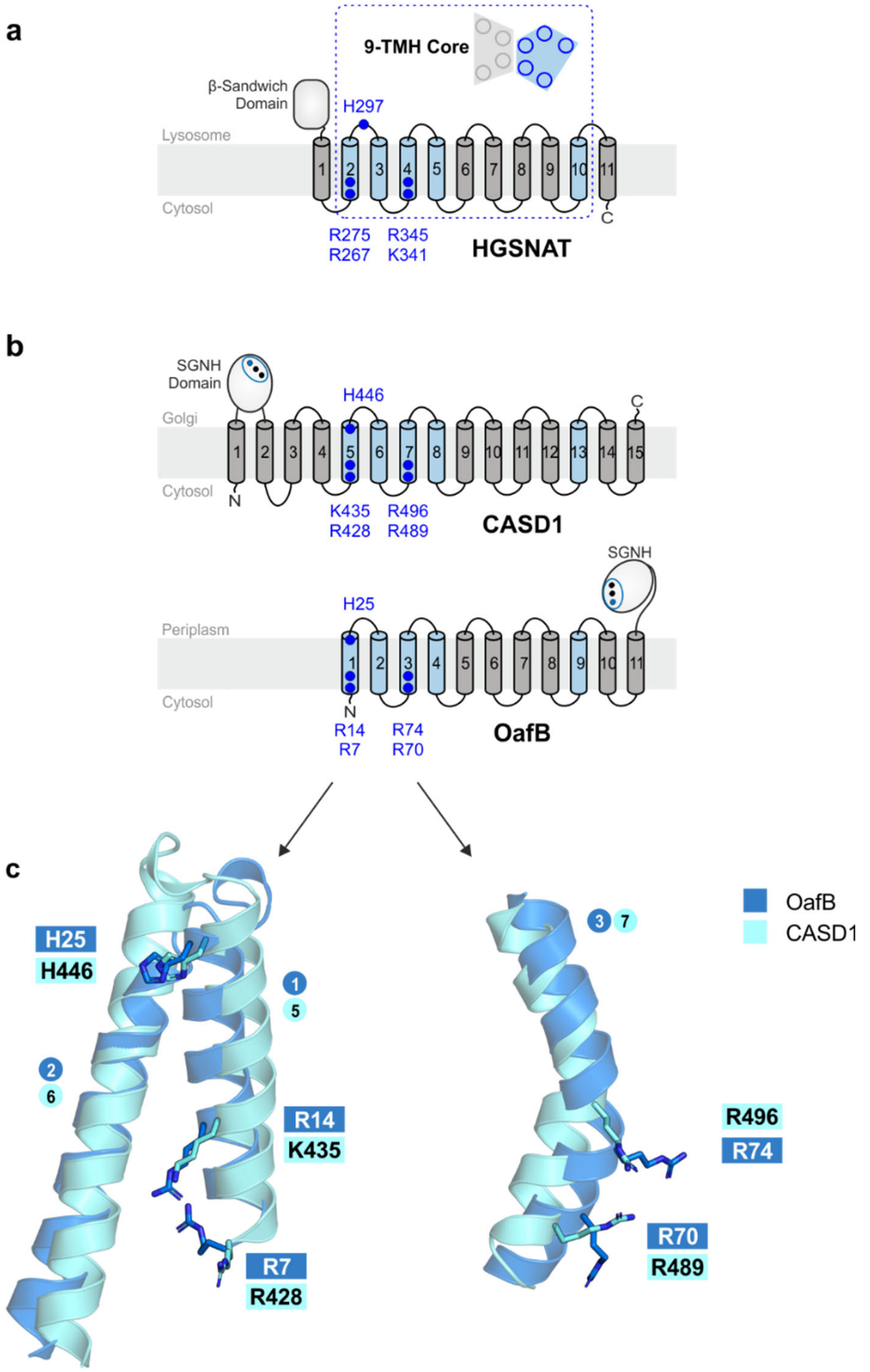

Supplementary Figure 23.

Structural conservation of positively charged amino acid residues. a, Scheme showing the location of the positively charged HGSNAT residues that are involved in acetyl-CoA binding[5, 6, 7]. TMHs forming the 9-TMH core are boxed, with TMHs of the catalytic core shown in blue. The amino acid numbering is based on the canonical UniProt sequence Q68CP4. **b,** Scheme showing the location of corresponding positively charged residues is CASD1 (see also Fig. 6i) and OafB. **c.** Enlarged details of the superposition of the AF2 models of CASD1 (cyan) and OafB (aquamarine). Cartoon representation with the indicated positively charged residues and the histidine critical for activity of CASD1 and OafB (see Fig. 6f,g and Ref. 8, respectively) shown in stick representation.

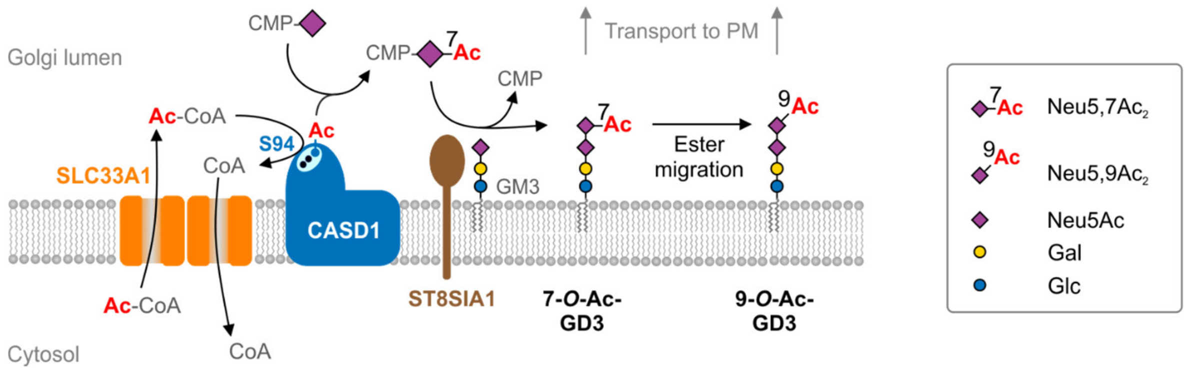

Supplementary Figure 24.

Proposed Model for the generation of 9-*O*-acetylated GD3. Based on prior evidence for 7-*O*-acetylated GD3 (7-*O*-Ac-GD3)[9, 10], we propose that ST8SIA1 preferentially utilizes 7-*O*-acetylated CMP-Sia—generated by the luminal catalytic domain (LCD) of CASD1—as a substrate, producing 7-*O*-Ac-GD3. Since the 7-*O*-acetyl group is prone to spontaneous migration to C9 of Sia, 7-*O*-Ac-GD3 is converted to 9-*O*-acetylated GD3 (9-*O*-Ac-GD3) over time[11]. This non-enzymatic process may take place within the Golgi apparatus or after transport to the plasma membrane (PM). According to this model, loss of SLC33A1 or inactivation of the CASD1 LCD through the S94A mutation disrupts the initial 7-*O*-acetylation step, thereby preventing the downstream production of 9-*O*-acetylated GD3.

Supplementary Tables

Supplementary Table 1. Disease-associated mutation in *SLC33A1* (Genbank accession NG_023365.1).

Huppke-Brendel Syndrome *(originally described as recessive cerebellar ataxia ATX-SLC33A1)*[12]

Lethal autosomal recessive disorder characterized by congenital cataract, hearing loss, developmental delay, hypomyelination, cerebellar hypoplasia, low copper and ceruloplasmin level; All patients affected by childhood-onset HBS died prematurely between ages 10 month and 6 years[13, 14, 15].

Mutation	*Protein Variant*	*Mode of inheritance*	*Description*	*Reference*
c.328 G>C	**p.Ala110Pro**	Recessive	Missense mutation in exon 1 (single case)	13
c.542_543delTG	p.Val181Glyfs*6	Recessive	2 bp deletion in exon 1 leading to a frameshift and premature stop codon (single case)	14
c.1098 C>G	**p.Tyr366***	Recessive	Nonsense mutation in exon 3 leading to a premature stop codon (single case)	13
c.1131C>G	p.Tyr377*	Recessive	Nonsense mutation in exon 3 leading to a premature stop codon (single case)	15
c.1267-1 G>A	n.d.	Recessive	Mutation of splice acceptor site of exon 5	13
c.[1474_1482+9del];<br>[615_616insT]<br>Compound heterozygous	n.d.<br>p.Leu205Phefs*31	Recessive	17 bp deletion affecting the 3'-end of exon 5 and the donor splice site in intron 5;<br>1 bp insertion in exon 1 leading to a frameshift and premature stop codon (single case)	13
c.[817_819del];<br>[1331T>C]<br>Compound heterozygous	p.Thr273del;<br>p.Ile444Thr	Recessive	First description of HBS in an adult (29 y); single case	16

Hereditary Spastic Paraplegia type 42 (SPG42)

Slowly progressive and bilateral spasticity of the legs; Spastic gait, increased muscle tone, hyperreflexia, and extensor plantar reflexes; Age at onset between 4 and 42 years[17, 18].

Mutation	Protein variant	Mode of inheritance	Description	Reference
c.339T>G	**p.Ser113Arg**	Dominant	Missense mutation in exon 1. Single family with 23 affected members known	17, 18

Late onset cerebellar ataxia

Slowly progressive spastic ataxic syndrome; Neurogenic bladder, spastic ataxic gait, brisk reflexes; Onset at 35 years[19].

Mutation	Protein variant	Mode of inheritance	Description	Reference
c.1525G>A	**p.Gly509Ser**	Dominant	Missense mutation in exon 6. Variant of uncertain significance (single case)<br>Originally reported as rs138283229, which has been merged to rs76440173 on July 19, 2016	19

Protein variants analyzed in this study are highlighted in blue.

Supplementary Table 2. Protein domain families assigned to the AT3 clan (Acyl_transf_3; CL0316). Data compiled from https://www.ebi.ac.uk/interpro/search/text/CL0316. The multi-TM domains of CASD1, HGSNAT and OafB, which have been analysed in this study, belong to the families PF07779 (Cas1_AcylT), PF07786 (HGSNAT_cat) and PF01757 (Acyl_transf_3), respectively.

**Accession**	**Short Name**	**Proteins**	**Taxonomy**
PF07786	HGSNAT_cat	>20.000	Bacteria, eukaryota, archaea
PF10129	OpgC_C	>6.000	Bacteria
PF16401	DUF5009	>2.000	Bacteria (mainly), eukaryota
PF07779	Cas1_AcylT	>5.000	Eukaryota
PF06423	GWT1	>5.000	Eukaryota
PF05857	TraX	>8.000	Bacteria (mainly), archaea
PF11318	DUF3120	>700	Bacteria (mainly), eukaryota
PF12291	DUF3623	>1.000	Bacteria
PF04235	DUF418	>16.000	Bacteria (mainly), archaea
PF01757	Acyl_transf_3	>138.000	Bacteria (mainly), eukaryota, archaea

DUF; domain of unknown function

Supplementary Table 3. Molecular dynamics simulations checklist.

**Reliability and reproducibility checklist for molecular dynamics simulations *All boxes must be marked YES by acceptance unless "Response not needed if No".**	**Yes**	**No**	**Response (Please state where this information can be found in the text)**
**1. Convergence of simulations and analysis**			
1a. Is an evaluation presented in the text to show that the property being measured has equilibrated in the simulations (*e.g.* time-course analysis)?	☒	☐	Computational methods and Supplementary Figure 18
1b. Then, is it described in the text how simulations are split into equilibration and production runs and how much data were analyzed from production runs?	☒	☐	Computational methods
1c. Are there at least 3 simulations per simulation condition with statistical analysis?	☒	☐	Computational methods and Supplementary Figure 18
1d. Is evidence provided in the text that the simulation results presented are independent of initial configuration?	☒	☐	Computational methods
**2. Connection to experiments**			
2a. Are calculations provided that can connect to experiments (*e.g.* loss or gain in function from mutagenesis, binding assays, NMR chemical shifts, J-couplings, SAXS curves, interaction distances or FRET distances, structure factors, diffusion coefficients, bulk modulus and other mechanical properties, *etc.*)?	☒	☐	Results section, paragraph describing MD-predicted substrate binding orientation and subsequent experimental validation (Fig. 6 and Supplementary Fig. 18).
**3. Method choice**			
3a. Do simulations contain membranes, membrane proteins, intrinsically disordered proteins, glycans, nucleic acids, polymers, or cryptic ligand binding?	☒	☐	Computational methods, Fig. 6 and Supplementary Fig. 18
3b. Is it described in the text whether the accuracy of the chosen model(s) is sufficient to address the question(s) under investigation (e.g. all-atom vs. coarse-grained models, fixed charge vs. polarizable force fields, implicit vs. explicit solvent or membrane, force field and water model, etc.)?	☒	☐	Computational methods

3c. Is the timescale of the event(s) under investigation beyond the brute-force MD simulation timescale in this study that enhanced sampling methods are needed?	☐	☒	
If **YES**, are the parameters and convergence criteria for the enhanced sampling method clearly stated?	☐	☐	
If **NO,** is the evidence provided in the text?	☒	☐	Computational methods
**4. Code and reproducibility**			
4a. Is a table provided describing the system setup that includes simulation box dimensions, total number of atoms, total number of water molecules, salt concentration, lipid composition (number of molecules and type)?	☒	☐	The system setup parameters are described in detail in the Methods Section (Computational methods)
4b. Is it described in the text what simulation and analysis software and which versions are used?	☒	☐	Computational methods
4c. Are other parameters for the system setup described in the text, such as protonation state, type of structural restraints if applied, nonbonded cutoff, thermostat and barostat, etc.?	☒	☐	Computational methods
4d. Are initial coordinate and simulation input files and a coordinate file of the final output provided as supplementary files or in a public repository?	☒	☐	These data are provided in the public repository Zenodo https://doi.org/10.5281/zenodo.18795789 See Reference 84 and Data Availability Statement
4e. Is there custom code or custom force field parameters?	☐	☒	
If **YES**, are they provided as supplementary files or in a public repository?	☐	☐	

Supplementary References

1. Zhou D., Chen N., Huang S., Song C., Zhang Z. Mechanistic insights into the acetyl-CoA recognition by SLC33A1. *Cell Discov.* **11**, 36 (2025).
2. Thompson J. D., Higgins D. G., Gibson T. J. CLUSTAL W: improving the sensitivity of progressive multiple sequence alignment through sequence weighting, position-specific gap penalties and weight matrix choice. *Nucleic Acids Res.* **22**, 4673-4680 (1994).
3. Corpet F. Multiple sequence alignment with hierarchical clustering. *Nucleic Acids Res.* **16**, 10881-10890 (1988).
4. Gouet, P., Courcelle, E., Stuart, D. I. and Metoz, F. (1999) "ESPript: multiple sequence alignments in PostScript". *Bioinformatics* **15**, 305-308.
5. Navratna V., Kumar A., Rana J. K., Mosalaganti S. Structure of the human heparan-α-glucosaminide N-acetyltransferase (HGSNAT). *Elife* **13**:RP93510 (2024).
6. Xu R., et al. Structure and mechanism of lysosome transmembrane acetylation by HGSNAT. *Nat. Struct. Mol. Biol.* **31**, 1502-1508 (2024).
7. Zhao B., et al. Structural and mechanistic insights into a lysosomal membrane enzyme HGSNAT involved in Sanfilippo syndrome. *Nat. Commun.* **15**, 5388 (2024).
8. Pearson C. R., et al. Acetylation of Surface Carbohydrates in Bacterial Pathogens Requires Coordinated Action of a Two-Domain Membrane-Bound Acyltransferase. *mBio* **11**:e01364-20 (2020).
9. Erdmann M., et al. Differential surface expression and possible function of 9-*O*- and 7-*O*-acetylated GD3 (CD60 b and c) during activation and apoptosis of human tonsillar B and T lymphocytes. *Glycoconj. J.* **23**, 627-638 (2006).
10. Arming S., et al. The human Cas1 protein: a sialic acid-specific *O*-acetyltransferase? *Glycobiology* **21**, 553–564 (2011).
11. Zhang Z., et al. Chemoenzymatically synthesized *O*-acetylated GD3 gangliosides to examine viral receptor specificities in a cellular context. *Angew. Chem. Int. Ed.* **65**:e17989 (2026)
12. Rossi M., et al. The genetic nomenclature of recessive cerebellar ataxias. *Mov. Disord.* **33**, 1056-1076 (2018).
13. Huppke P., et al. Mutations in SLC33A1 cause a lethal autosomal-recessive disorder with congenital cataracts, hearing loss, and low serum copper and ceruloplasmin. *Am. J. Hum. Genet.* **90**, 61-68 (2012).
14. Chiplunkar S., et al. Huppke-Brendel syndrome in a seven months old boy with a novel 2-bp deletion in SLC33A1. *Metab. Brain. Dis.* **31**, 1195-1198 (2016).
15. Šikić K., et al. Abnormal concentrations of acetylated amino acids in cerebrospinal fluid in acetyl-CoA transporter deficiency. *J. Inherit. Metab. Dis.* **45**, 1048-1058 (2022).
16. Kirk F. T., et al. Case report: Huppke-Brendel syndrome in an adult, mistaken for and treated as Wilson disease for 25 years. *Front. Neurol.* **13**, 957794 (2022).
17. Lin P., et al. A missense mutation in SLC33A1, which encodes the acetyl-CoA transporter, causes autosomal-dominant spastic paraplegia (SPG42). *Am. J. Hum. Genet.* **83**, 752-759 (2008).
18. Mao F., et al. Identification and functional analysis of a SLC33A1: c.339T>G (p.Ser113Arg) variant in the original SPG42 family. *Hum. Mutat.* **36**, 240-249 (2015).
19. Keogh M. J., et al. Frequency of rare recessive mutations in unexplained late onset cerebellar ataxia. *J. Neurol.* **262**, 1822-1827 (2015).
