# Supplementary material for: Interplay of SLC33A1-dependent and -independent Golgi sialic acid O-acetylation in CASD1 catalysis
Source: Nat Commun. 2026 Apr 1;17:3156. doi: 10.1038/s41467-026-71333-y (PMC13043746; doi:10.1038/s41467-026-71333-y)
Supplement: Supplementary file 2 — Description of Additional Supplementary Files [file 41467_2026_71333_MOESM2_ESM.pdf]

Description of Additional Supplementary Files

File Name: Supplementary Data 1

Description: The data set contains a list of the expression plasmids and CRISPR/Cas plasmids generated in this study and information on the sequences of primers and oligonucleotides used for their generation.
