# Supplementary material for: Interplay of SLC33A1-dependent and -independent Golgi sialic acid O-acetylation in CASD1 catalysis
Source: Nat Commun. 2026 Apr 1;17:3156. doi: 10.1038/s41467-026-71333-y (PMC13043746; doi:10.1038/s41467-026-71333-y)
Supplement: Supplementary file 4 — Reporting Summary [file 41467_2026_71333_MOESM4_ESM.pdf]

nature portfolio

Corresponding author(s): Martina Mühlenhoff

Last updated by author(s): Mar. 01, 2026

Reporting Summary

Nature Portfolio wishes to improve the reproducibility of the work that we publish. This form provides structure for consistency and transparency in reporting. For further information on Nature Portfolio policies, see our Editorial Policies and the Editorial Policy Checklist.

Statistics

For all statistical analyses, confirm that the following items are present in the figure legend, table legend, main text, or Methods section.

n/a	Confirmed	
☐	☒	The exact sample size (*n*) for each experimental group/condition, given as a discrete number and unit of measurement
☐	☒	A statement on whether measurements were taken from distinct samples or whether the same sample was measured repeatedly
☐	☒	The statistical test(s) used AND whether they are one- or two-sided<br>*Only common tests should be described solely by name; describe more complex techniques in the Methods section.*
☒	☐	A description of all covariates tested
☐	☒	A description of any assumptions or corrections, such as tests of normality and adjustment for multiple comparisons
☐	☒	A full description of the statistical parameters including central tendency (e.g. means) or other basic estimates (e.g. regression coefficient) AND variation (e.g. standard deviation) or associated estimates of uncertainty (e.g. confidence intervals)
☐	☒	For null hypothesis testing, the test statistic (e.g. *F*, *t*, *r*) with confidence intervals, effect sizes, degrees of freedom and *P* value noted<br>*Give P values as exact values whenever suitable.*
☒	☐	For Bayesian analysis, information on the choice of priors and Markov chain Monte Carlo settings
☒	☐	For hierarchical and complex designs, identification of the appropriate level for tests and full reporting of outcomes
☒	☐	Estimates of effect sizes (e.g. Cohen's *d*, Pearson's *r*), indicating how they were calculated

Our web collection on statistics for biologists contains articles on many of the points above.

Software and code

Policy information about availability of computer code

Data collection	MD simulations were performed using the GPU verson of GROMACS 2022 (https://www.gromacs.org/). Force field topology and parameters for MD simulations were obtained from the GROLIGFF web-server (v1.0, https://groligff.net/).
Data analysis	LC-MS raw data were processed using TraceFinder (v5.0.889.0, Thermo Fisher Scientific); Immunofluorescence microscopy images were processed using the ZEN software (v2012, Zeiss); PyMOL (v2.3, DeLano Scientific) was used for visual inspection of structural models and graphical images of protein structures. FlowJo software (v7.6, FlowJo, LCC) was used to analyse and visualize the flow cytometric data. Prism software (v8, GraphPad) was used for statistical analyses.

For manuscripts utilizing custom algorithms or software that are central to the research but not yet described in published literature, software must be made available to editors and reviewers. We strongly encourage code deposition in a community repository (e.g. GitHub). See the Nature Portfolio guidelines for submitting code & software for further information.

Data

Policy information about availability of data

All manuscripts must include a data availability statement. This statement should provide the following information, where applicable:

- Accession codes, unique identifiers, or web links for publicly available datasets
- A description of any restrictions on data availability
- For clinical datasets or third party data, please ensure that the statement adheres to our policy

The molecular dynamics simulations data generated in this study (starting structure, force field parameter files, molecular dynamics parameter and trajectories)

have been deposited in the Zenodo database (https://doi.org/10.5281/zenodo.18795789). The source data underlying Figs. 2b-c, 2e-f, 3a-b, 4d, 4f, 4h, 5d, 6f-g, and Supplementary Figs. 6, 11 and 19 are provided as a Source Data file. Source data are provided with this paper.

Research involving human participants, their data, or biological material

Policy information about studies with human participants or human data. See also policy information about sex, gender (identity/presentation), and sexual orientation and race, ethnicity and racism.

Reporting on sex and gender	N/A
Reporting on race, ethnicity, or other socially relevant groupings	N/A
Population characteristics	N/A
Recruitment	N/A
Ethics oversight	N/A

Note that full information on the approval of the study protocol must also be provided in the manuscript.

Field-specific reporting

Please select the one below that is the best fit for your research. If you are not sure, read the appropriate sections before making your selection.

[x] Life sciences [ ] Behavioural & social sciences [ ] Ecological, evolutionary & environmental sciences

For a reference copy of the document with all sections, see nature.com/documents/nr-reporting-summary-flat.pdf

Life sciences study design

All studies must disclose on these points even when the disclosure is negative.

Sample size	Sample sizes were selected based on prior experience and field standards (at least three biological replicates), and were sufficient to detect reproducible, biologically meaningful differences; no formal calculation was performed.
Data exclusions	No data were excluded from the analyses.
Replication	All key experiments were performed at least three times, including independent biological replicates. The exact number of independent experiments and the number of technical replicates for each experiment are provided in the figure legends. Results were reproducible across independent experiments.
Randomization	No randomization was perfomed. No covariates were included, as the experiments were conducted in controlled in vitro systems with predefined experimental groups.
Blinding	Blinding was not relevant, as all statistical analyses were based on quantitative, instrument-generated data (flow cytometry and in vitro assay readouts) acquired using predefined settings and analyzed with standardized pipelines. Immunoblots, immunofluorescence microscopy images and immuno-TLCs are presented as representative experiments and were not subject to subjective scoring.

Reporting for specific materials, systems and methods

We require information from authors about some types of materials, experimental systems and methods used in many studies. Here, indicate whether each material, system or method listed is relevant to your study. If you are not sure if a list item applies to your research, read the appropriate section before selecting a response.

Materials & experimental systems

n/a	Involved in the study	
[ ]	[x]	Antibodies
[ ]	[x]	Eukaryotic cell lines
[x]	[ ]	Palaeontology and archaeology
[x]	[ ]	Animals and other organisms
[x]	[ ]	Clinical data
[x]	[ ]	Dual use research of concern
[x]	[ ]	Plants

Methods

n/a	Involved in the study	
[x]	[ ]	ChIP-seq
[ ]	[x]	Flow cytometry
[x]	[ ]	MRI-based neuroimaging

Antibodies

Antibodies used

Primary antibodies:

Anti-9-O-Ac-GD3 mAb (Santa Cruz cat. Sc 32269, clone UM4D4); anti-9-O-Ac-GD3 mAb (Ancell cat. ANC-212-820, clone UM4D4); anti 9-O-Ac GD3 mAb (Thermo Scientific cat. MA1 34707, clone M-T6004); anti-GD3 mAb R24 (purified in-house by protein A affinity chromatography from cell culture supernatant of hybridoma cells ATCC HB-8445); anti-9-O-Ac-GD2 mAb 8B6 (kindly provided by OGD2 Pharma, Nantes); anti-GD2 mAb (Kerafast cat. EWI023, clone ME361); anti-Flag pAb (DYKDDDDK-Tag antibody; Cell Signaling cat. 2368); anti-Flag mAb (Sigma-Aldrich cat. F1804, clone M2); anti-Myc mAb (ThermoFisher cat. MA1-980, clone 9E10); anti-V5 mAb (Acris cat. SM1691PS, clone SV5-PK1); chicken anti-V5 pAb (Abcam cat. ab9113); anti-α-Mannosidase II pAb (kindly provided by Kelley Moremen); anti-actin mAb (Merck Millipore cat. MAB1501, clone C4); anti-Actin pAb (1:1000; Sigma-Aldrich cat. A2066).

Secondary antibodies:

Goat anti-chicken IgY Alexa Fluor 488-conjugate (Invitrogen cat. A 11039), donkey anti-human IgG Dylight 550-conjugate (Invitrogen cat. SA5 10127), goat anti-mouse IgG Alexa Fluor 488-conjugate (Invitrogen cat. A-11029), sheep anti-mouse IgG Cy3-conjugate (Sigma-Aldrich cat. C2181), goat anti-mouse IgG2a Alexa Fluor 488-conjugate (Invitrogen cat. A-21131), rabbit anti-mouse IgG3 DyLight 549-conjugate (Rockland cat. 610-442-043), goat anti-mouse IgM Alexa Fluor 568-conjugate (Invitrogen cat. A 21043), goat anti-rabbit IgG Alexa Fluor 488-conjugate (Invitrogen cat. A-11008), sheep anti-rabbit IgG Cy3-conjugate (Sigma-Aldrich cat. C2306); goat anti-mouse IgG IRDye 800CW-conjugate (LI-COR Biosciences cat. 926 32210); goat anti-mouse IgM IRDye 800CW-conjugate (LI-COR Biosciences cat. 926 32280); anti-rabbit IgG IRDye 800CW-conjugate (LI-COR Biosciences cat. 926 32211); goat anti-rabbit IgG horseradish peroxidase-conjugate (Sigma-Aldrich cat. A6154); goat anti-mouse IgG POD-conjugate (SouthernBiotech cat. 1010-05); rat anti-mouse IgM PE-conjugate (BD cat. 553409) or goat anti-human IgG PE-conjugate (Jackson ImmunoResearch, cat. 109-115-098); donkey anti-mouse IgG Alexa 555 (Invitrogen cat. A-31570).

Validation

The antibodies UM4D4 (Kniep et al. 1992. Biochem. Biophys. Res. Commun. 187:1343-1349), M-T6004 (Kniep et al. 2006. Int. J. Cancer 119:67-73), R24 (Dippold et al. 1980. Proc. Natl. Acad. Sci. USA 77::6114-6118; Houghton et al. 1985. Proc. Natl. Acad. Sci. USA 82:1242-1246), 8B6 (Cerato et al. 1997. Hybridoma 16:307-316) and ME361 (Ahmed and Cheung 2014. FEBS Lett. 588:288-297) recognize mammalian ganglioside-derived glycan structures. In the present study, their specificity has been verified by immuno-TLC on purified gangliosides and by the absence of binding to ganglioside extracts from mock-transfected cells lacking the glycosyltransferase(s) required for the biosynthesis of the respective ganglioside. The selectivity of the antibodies UM4D4 and M-T6004 for the 9-O-acetylated form of GD3 and of 8B6 for 9-O-Ac-GD2 has been previously validated by enzymatic removal of the 9-O-acetyl group (Albers et al. 2021. Glycobiology 31:1176–1191) and by the absence of binding to GD3 and GD2 extracted from O-acetylation deficient, CASD1 knockout cells (Baumann et al. 2015. Nat. Commun. 6:7673; Cavdarli et al. 2021. Cells 10:1468).

The specificity of the anti-tag antibodies has been validated by the absence of binding to mock-transfected cells, lacking epitope-tagged proteins, or by Western blot analysis of protein extracts of mock-transfected cells.

The anti-α-mannosidase II pAb is a well characterized Golgi marker antibody that recognizes the luminal catalytic domain of Golgi α-mannosidase II in rat, mouse, human and hamster (CHO) cells (Moremen et al. 1991. J. Biol. Chem., 266:16876-16885; Velasco et al. 1993. J. Cell Biol. 122:39-51).

The anti-actin mAb (clone C4), which was used in this study to detect actin as a loading control for tWestern blot analyses (Fig. 3b and Supplementary Fig. 6), is a highly published pan-actin antibody (see MAB1501 at https://www. Merckmilipore.com) that binds to an epitope in a highly conserved region of actin, which allows the detection of all six isoforms of vertebrate actin (Lessard 1988. Cell Motil Cytoskeleton. 10:349-62).

The binding specificities of the virolectins ICV-HE0-Fc and BCoV-HE0-Fc was previously characterized in detail on a sialoglycan array (Li et al. 2021. Nat. Chem. 13: 496-503) and by the loss of binding to cellular sialoglycans upon selective removal of O-acetyl groups from sialic acids (Albers et al. 2021. Glycobiology 31:1176–1191). In the present study, the specificity has been validated by the absence of binding to sialoglycans displayed by O-acetylation deficient, CASD1 knockout cells.

Eukaryotic cell lines

Policy information about cell lines and Sex and Gender in Research

Cell line source(s)

HAP1 cells (RRID: CVCL_Y019) and HAP1-ΔSLC33A1 cells (HAP1_SLC33A1_36819-02) were purchased from Haplogen Genomics (Austria). CHO-K1 cells were obtained from the Institute of Medical Microbiology, Hannover Medical School, Hannover, Germany (Eckhardt et al. 1995 Nature 373, 715–718). CHO-ΔCasd1 cells have been generated previously (Cavdarli et al. 2021 Cells 10, 1468) and CHO-ΔSlc33a1 cells have been generated in this study.

Authentication

HAP1 cells (parental and ΔSLC33A1) were authenticated as human cells of the indicated genotype by sequencing part of the SLC33A1 locus. CHO-K1 cells (parental, ΔCasd1 and ΔSlc33a1) were authenticated by sequencing part of the Slc33a1 and the Casd1 locus followed by sequence comparison with the CHO-K1 genome (https://chogenome.org/).

Mycoplasma contamination

All cell lines were tested negative for mycoplasma.

Commonly misidentified lines
(See ICLAC register)

Name any commonly misidentified cell lines used in the study and provide a rationale for their use.

Plants

Seed stocks	N/A
Novel plant genotypes	N/A
Authentication	N/A

Flow Cytometry

Plots

Confirm that:

- [x] The axis labels state the marker and fluorochrome used (e.g. CD4-FITC).
- [x] The axis scales are clearly visible. Include numbers along axes only for bottom left plot of group (a 'group' is an analysis of identical markers).
- [x] All plots are contour plots with outliers or pseudocolor plots.
- [x] A numerical value for number of cells or percentage (with statistics) is provided.

Methodology

Sample preparation	As only cultured cells were analysed, no specific sample preparation was required.
Instrument	CyFlow ML flow ytometer (Sysmex Partec) equipped with a 488 nm laser.
Software	FlowJo software version 7.6
Cell population abundance	Since we analyzed only defined cell culture lines, cell sorting was not required. The abundance of cells that displayed O-acetylated sialoglycans is given as "% Positive cells".
Gating strategy	The gating strategies used to determine positive cells are shown in the Supplementary Information. Mock-transfected cells stained with secondary antibody only and/or fully stained mock-transfected cells were used to guide the gating strategy.

- [x] Tick this box to confirm that a figure exemplifying the gating strategy is provided in the Supplementary Information.
